# Hierarchical YOLO26 with Axis-Wise Context Block and Poincaré Supervision for Fine-Grained Ship Detection in Remote Sensing

**DOI:** 10.3390/s26206584

**Published:** 2026-10-16

**Authors:** Yahya İzala, Yaşar Becerikli

**Affiliations:** 1 Research and Development Unit, Derince District Directorate of National Education, Kocaeli 41900, Türkiye; 2 Digital Forensics Department, The Council of Forensic Medicine (ATK), Istanbul 34184, Türkiye; ybecerikli@kocaeli.edu.tr; 3 Department of Computer Engineering, Faculty of Engineering, Kocaeli University, Kocaeli 41100, Türkiye

**Keywords:** oriented object detection, hierarchical ship recognition, YOLO26, Poincaré supervision, axis-wise context block, remote sensing

## Abstract

**Highlights:**

**What are the main findings?**

- Assignment-aligned parent targets and training-time Poincaré path supervision integrate the ship taxonomy into a dense YOLO oriented detector while preserving native leaf-score ranking at inference.
- Gated axis-wise filtering before pyramid fusion and local-window Feature Guidance provide complementary context and cross-level semantic interaction.

**What are the implications of the main findings?**

- Explicit taxonomic supervision can be effectively integrated into a dense single-stage oriented detector to enhance fine-grained semantic discrimination while preserving the detector’s native leaf-class inference pathway and avoiding inference-time hyperbolic score fusion.
- The accuracy gains are accompanied by substantial computational overhead, indicating that model compression, Feature Guidance optimization, and deployment-oriented acceleration are important directions for practical real-time applications.

**Abstract:**

Fine-grained oriented ship detection remains challenging because visually similar vessel categories may exhibit substantial variations in scale, aspect ratio, and orientation. To address these challenges, this study proposes YOLO26-OBB-HMS (Hierarchical Multi-Label Supervision), a hierarchy-aware extension of YOLO26-OBB that integrates a pre-pyramid Axis-wise Context Block, local-window Feature Guidance, assignment-aligned parent-level supervision, and training-time Poincaré path learning while preserving the detector’s native oriented-box formulation and leaf-level inference scoring. Under the common class-wise AP50 evaluation protocol, the proposed n- and s-scale variants achieved 77.5% and 82.7% on the filtered HRSC2016 test partition and 75.4% and 80.6% on the prepared ShipRSImageNet final-evaluation partition, respectively.

## 1. Introduction

Fine-grained ship detection in optical remote-sensing imagery requires the simultaneous localization of vessels and discrimination among visually similar ship categories. Unlike generic object detection, this task is complicated by large variations in object scale, aspect ratio, and orientation, as well as by the subtle structural differences separating closely related vessel types. Ships may occupy only a small portion of a high-resolution image, appear at arbitrary angles, or be partially obscured by complex maritime backgrounds. Harbors, coastlines, docks, waves, and densely arranged vessels can further introduce visual patterns that resemble ship structures, increasing both localization and classification difficulty [1,2,3].

Oriented object detection has considerably improved the geometric modeling of such targets. Two-stage approaches such as Oriented R-CNN employ rotated proposals and orientation-aware region features, whereas single-stage detectors such as R^3^Det and S^2^A-Net progressively refine or align features for arbitrarily oriented objects [4,5,6]. More recently, YOLO26-OBB has provided a dense end-to-end framework for oriented detection while preserving the computational simplicity of the YOLO family [7]. These methods are effective in estimating object location, extent, and orientation; however, their semantic prediction is generally formulated as flat classification over leaf categories. Under this formulation, all incorrect classes are treated as unrelated alternatives, although fine-grained ship labels commonly follow an explicit taxonomy. Consequently, a confusion between two closely related destroyer types is penalized in the same manner as a confusion between semantically distant vessel categories.

Hierarchical supervision provides a principled means of exploiting these taxonomic relationships during representation learning. By incorporating labels at multiple semantic levels, a detector can learn both the shared characteristics of broad vessel groups and the subtle features required to distinguish their subordinate classes. Hierarchy-aware learning has been shown to encourage semantically more meaningful representations and errors [8]. In fine-grained oriented ship detection, Hierarchical Multi-Label Ship Detection Network (HMS-Net) introduced multi-level representation learning, Feature Guidance, and a hierarchy-aware relationship constraint within a two-stage detection architecture [9]. Nevertheless, directly transferring this strategy to a dense YOLO detector is not straightforward. HMS-Net operates on compact proposal-wise rotated RoI features, whereas a single-stage detector predicts objects from multi-scale dense feature maps. Hierarchical targets must therefore remain consistent with the foreground locations selected by the host detector, and cross-level semantic interaction must be implemented without incurring the prohibitive cost of global attention over high-resolution maps.

A related challenge arises from the elongated geometry of ships. Conventional isotropic convolution may be insufficient to capture long-range structural patterns along vessel hulls, particularly when the targets exhibit extreme aspect ratios or occupy only a small image region. Selective large kernels [10], poly-kernel feature extraction [11], and strip convolutions [12] have demonstrated the effectiveness of anisotropic receptive fields in remote-sensing object detection. These findings motivate the use of axis-wise context before multi-scale feature fusion. However, horizontal and vertical filtering in the image coordinate system should not be confused with explicit orientation alignment. Such filtering can enrich the anisotropic context, but it does not rotate the representation into a vessel-aligned coordinate frame or guarantee rotational equivariance.

The representation of the taxonomy itself introduces an additional methodological consideration. Euclidean spaces may require comparatively high dimensionality to represent tree-like relationships because the number of nodes can grow rapidly with taxonomic depth. Hyperbolic spaces, particularly the Poincaré ball, provide a suitable geometry for embedding hierarchical structures because their available volume increases exponentially with distance from the origin [13]. Hyperbolic representations have also been investigated in hierarchical multi-label classification [14]. For dense fine-grained ship detection, however, taxonomy prototypes alone are insufficient: the visual embeddings associated with foreground predictions must also be aligned with the corresponding leaf classes and their ancestors. Moreover, if hyperbolic scores are combined directly with native detection confidences during inference, any observed improvement may be influenced by an additional score-fusion or calibration mechanism rather than by representation learning alone.

To address these limitations, this study proposes YOLO26-OBB-HMS, a hierarchy aware extension of YOLO26-OBB for fine-grained oriented ship detection. The framework integrates axis-wise context modeling, local cross-level Feature Guidance, assignment aligned parent supervision, and training-time Poincaré path supervision within a unified dense detector. An Axis-wise Context Block (ACB) is applied to the backbone lateral features before Feature Pyramid Network (FPN) or Path Aggregation Network (PAN) fusion, allowing anisotropic information to propagate into both the semantic and localization pathways. At each detection scale, computationally bounded Feature Guidance modules facilitate interaction among coarse, intermediate, and leaf-level representations through local window cross-attention. Parent targets are derived from the same foreground assignments used by the native leaf detector, thereby maintaining consistency between hierarchical supervision and dense object assignment.

The geometric and semantic pathways are deliberately separated. Native box-distance and angle prediction towers operate on the neck features, whereas the native leaf classification tower uses the refined semantic representation. Coarse- and intermediate level classifiers, visual embeddings, and hyperbolic path scores serve as auxiliary training signals. During inference, detections are ranked exclusively using the native YOLO leaf class scores; parent predictions and Poincaré distances neither replace nor re-rank these scores. This design enables the influence of hierarchical representation learning to be evaluated without introducing a new oriented-box regression loss, hard taxonomic routing, or an inference-time score-fusion rule. The taxonomy regularizer encourages parent–child proximity and separation between unrelated prototypes, but it is not presented as guaranteeing radial depth ordering or an order-preserving hyperbolic embedding.

The principal contributions of this study are summarized as follows:

A dense hierarchy-aware formulation of YOLO26-OBB is developed in which coarse, intermediate, and leaf supervision follows the foreground assignments of the host detector. Local-window Feature Guidance enables computationally tractable cross-level semantic interaction on multi-scale feature maps while retaining the native oriented-box prediction pathway.

A pre-FPN Axis-wise Context Block is introduced to aggregate parallel horizontal and vertical depthwise responses through input-adaptive gating. The module enriches anisotropic contextual information before pyramid fusion and is explicitly distinguished from rotation alignment or rotationally equivariant feature extraction.

A training-time hyperbolic supervision strategy is formulated by aligning assigned visual embeddings with learnable leaf and ancestor prototypes in a Poincaré ball and regularizing the resulting taxonomy structure. The hyperbolic branch remains auxiliary during inference, allowing improvements in native leaf-level detection to be attributed to representation learning rather than to hyperbolic score fusion.

The proposed framework is evaluated on HRSC2016 and ShipRSImageNet using both n- and s-scale YOLO26-OBB models. The experimental analysis includes component-wise ablations, matched controls for axis-wise context and hyperbolic supervision, classwise comparisons with representative oriented detectors, hierarchical classification measurements, and computational-efficiency analysis. The results show that the proposed hierarchy-aware formulation improves aggregate fine-grained detection performance over the corresponding native YOLO26-OBB models, although the additional Feature Guidance and auxiliary semantic components introduce substantial computational overhead. Accordingly, the present framework is positioned as an accuracy-oriented formulation, while lightweight deployment optimization remains an important direction for future research.

The remainder of this paper is organized as follows. Section 2 reviews related studies on oriented object detection, axis-wise feature extraction, hierarchical ship recognition, and hyperbolic representation learning. Section 3 presents the proposed architecture, hierarchical supervision mechanism, Poincaré objectives, and inference pathway. Section 4 describes the experimental protocol and discusses the results. Finally, Section 5 concludes the study and outlines directions for future work.

## 2. Related Studies

Oriented object detection provides the geometric basis for recognizing ships in high-resolution remote-sensing imagery, where targets frequently exhibit arbitrary orientations, elongated shapes, large aspect-ratio variations, and dense spatial distributions. Unlike horizontal detectors, rotated detection methods must additionally cope with angular periodicity, boundary discontinuities, and the misalignment between axis-aligned convolutional features and oriented targets. Oriented R-CNN addresses these challenges through a two-stage framework that combines an Oriented Region Proposal Network with rotation-aware RoI feature extraction [4]. In the single-stage setting, R^3^Det progressively refines rotated bounding boxes while reconstructing more accurately aligned features [5], whereas S^2^A-Net explicitly aligns convolutional features with oriented objects through feature- and anchor-alignment mechanisms [6]. Alternative localization formulations have approached the same problem from a geometric-loss perspective. The Gaussian Wasserstein Distance represents rotated boxes as Gaussian distributions to alleviate sensitivity to angular discontinuities [15], while Kullback–Leibler Divergence-based regression adapts localization optimization according to object geometry and the aspect ratio [16]. Collectively, these methods substantially improve oriented localization, but their primary objective remains geometric detection rather than exploiting semantic relationships among fine-grained object categories.

Recent YOLO-based oriented detectors provide an efficient single-stage alternative for arbitrarily oriented ship detection in remote-sensing imagery [17]. In such architectures, classification and localization are performed directly over multi-scale dense feature maps rather than over independently generated rotated RoI features. This distinction is particularly important when introducing hierarchical semantic supervision: parent- and fine-level targets must be associated with the same foreground assignments used by the detector instead of being attached to independently sampled proposals. From this perspective, task-aligned learning approaches such as TOOD [18], which jointly consider classification and localization quality during foreground assignment, provide useful conceptual background for assignment-aware semantic supervision. The complete TOOD architecture, however, is not required for exploiting this principle in a dense oriented detector.

A complementary research direction concerns the enlargement and adaptation of receptive fields for objects whose spatial structures are strongly anisotropic. Inception-style architectures established the effectiveness of combining parallel convolutional branches with different spatial extents [19]. More recent remote-sensing detectors extend this principle using large or shape-adaptive kernels. LSKNet employs dynamically selected large kernels to capture broad contextual information in aerial imagery [10], while PKINet integrates poly-kernel depthwise convolutions with contextual attention to represent objects at different spatial scales [11]. Strip R-CNN further demonstrates the effectiveness of large strip convolutions for elongated remote-sensing objects [12]. These approaches are particularly relevant to ship detection because vessel structures are commonly characterized by long longitudinal extents and substantially shorter transverse dimensions. They therefore motivate the use of direction-sensitive contextual representations rather than relying exclusively on isotropic convolutional receptive fields.

It is nevertheless important to distinguish directional context modeling from explicit orientation alignment. Methods such as S^2^A-Net are designed to geometrically align feature representations with rotated objects [6], whereas strip-kernel approaches mainly adapt the receptive field to elongated spatial structures [12]. This distinction is relevant to the Axis-wise Context Block considered in the present work: rather than rotating or explicitly aligning features, parallel 1 × *k* and *k* × 1 depthwise responses capture complementary horizontal and vertical contextual patterns before multi-scale FPN/PAN fusion. Thus, the underlying motivation is closer to anisotropic contextual aggregation than to explicit rotation-aligned feature extraction.

Ship-specific detection studies further demonstrate the importance of contextual modeling, feature preservation, and multi-scale fusion under complex maritime conditions. Lightweight convolutional architectures have been investigated for multi-source infrared ship detection, while deep feature representations combined with conventional classifiers have also been explored for ship-type recognition. Previous hierarchical ship-detection research has additionally shown the value of jointly considering localization and semantic category structure under degraded imaging conditions. More recently, CPDF-Net combines Laplacian-pyramid decomposition, anisotropic filtering, contextual attention, and cascaded feature fusion for SAR ship detection [20]. Han et al. enhance YOLOv8 through semantic feature enhancement, lightweight multi-scale fusion, and improved localization optimization for multi-ship detection and classification [21], whereas Mazen and Mazen employ a two-stage pipeline that combines ship classification with instance segmentation in satellite imagery [22]. Although these studies improve robustness to scale variation, clutter, and difficult maritime backgrounds, most still formulate ship categories as independent flat classes and do not explicitly exploit the internal semantic taxonomy among fine-grained vessel types.

This limitation becomes increasingly important in fine-grained ship recognition. Closely related vessel categories often share similar hull structures, superstructures, and visual appearances, making errors between semantically neighboring classes difficult to avoid. Reviews of SAR-based ship classification identify limited data availability, feature representation, transfer learning, and discrimination among similar classes as recurring challenges [23]. Related maritime classification studies likewise report strong overall recognition performance while highlighting the difficulty of distinguishing visually similar civilian and military vessel categories [24]. At the dataset level, High-Resolution Ship Collections 2016 (HRSC2016) provides oriented ship annotations together with multi-level category information [25], whereas ShipRSImageNet offers a substantially larger fine-grained remote-sensing benchmark organized according to a multi-level ship taxonomy [26]. Such datasets naturally motivate learning strategies that exploit semantic relationships beyond independent leaf-level classification.

Hierarchical classification provides a principled framework for incorporating these relationships. Bertinetto et al. demonstrate that known class hierarchies can be exploited during learning so that semantically closer mistakes are treated differently from errors between distant categories [8]. Hierarchical multi-label methods further emphasize the importance of consistency across semantic levels. In particular, C-HMCNN introduces explicit mechanisms for maintaining coherence between parent and child predictions [27]. These two ideas should be distinguished conceptually. Multi-level supervision can shape intermediate feature representations by providing learning signals at several levels of a taxonomy, whereas probabilistic coherence requires an additional constraint ensuring that predictions at different levels satisfy predefined hierarchical relationships. A model may therefore benefit from hierarchical supervision without necessarily imposing hard or probabilistic cross-level consistency during inference.

Among existing approaches, HMS-Net is particularly relevant to fine-grained oriented ship detection because it explicitly incorporates hierarchical information into a rotated detection framework [9]. HMS-Net introduces Multi-scale Region Feature Re-extraction (MRFR), Feature Guidance across semantic levels, and a hierarchical relationship constraint over learned representations. Its evaluation on HRSC2016 and ShipRSImageNet demonstrates the potential of hierarchy-aware representation learning for fine-grained ship recognition [9]. However, HMS-Net is built around two-stage rotated detection and operates on compact proposal-wise rotated RoI features. Consequently, its hierarchical components cannot be transferred directly to a single-stage dense detector, where predictions originate from multi-scale feature maps and semantic supervision must remain synchronized with dense foreground assignment. Adapting hierarchy-aware learning to this setting therefore requires reconsidering both feature interaction and target routing.

Metric learning provides another mechanism for organizing semantically related categories. Proxy Anchor Loss represents each class through a learnable proxy and optimizes relationships between these proxies and sample embeddings, providing an efficient alternative to exhaustive sample-pair construction [28]. HMS-Net incorporates a proxy-based formulation into its hierarchical relationship modeling [9]. Such Euclidean proxy objectives provide a useful baseline for hierarchy-aware representation learning; however, they do not inherently encode the geometric expansion characteristic of tree-structured taxonomies.

Cross-level semantic interaction also introduces computational challenges when moving from proposal-wise features to dense multi-scale feature maps. Window-based attention provides an established mechanism for restricting attention computation to spatially local regions. Swin Transformer, for example, performs self-attention within local windows and introduces shifted windows to enable interaction across neighboring regions [29]. The relevance of this principle to dense hierarchical detection lies primarily in computational locality rather than in reproducing the Swin architecture itself. Local window-based feature interaction can provide semantic communication between hierarchy levels while avoiding the quadratic cost associated with global attention over high-resolution feature maps.

Hyperbolic representation learning offers a complementary solution for modeling hierarchical semantic structure. Poincaré embeddings introduced by Nickel and Kiela demonstrate that negatively curved spaces can represent tree-like relationships compactly, with rapidly increasing representational capacity toward the boundary of the space [13]. Hyperbolic models have subsequently been extended to hierarchical multi-label classification; for example, the Hyperbolic Interaction Model jointly exploits hierarchical label structure and instance representations in hyperbolic space [14]. These properties make Poincaré geometry attractive for ship taxonomies in which coarse categories progressively branch into increasingly fine-grained vessel types.

The applicability of hyperbolic representations has also been examined in object-detection settings. Lang et al. investigate hyperbolic embeddings and classifiers across object-detection architectures [30], while Kong et al. incorporate hyperbolic representation learning into open-world detection using vision–language information [31]. These studies indicate that non-Euclidean representations can be integrated with detection frameworks, but their objectives differ from taxonomy-supervised fine-grained ship detection. In the latter case, the central problem is not simply to replace a Euclidean detector classifier with a hyperbolic one, but to use a known ship taxonomy to regularize detector-selected visual embeddings while preserving the native detection pathway.

The manner in which hierarchical geometry is regularized is also important. Hyperbolic entailment cones explicitly model directed partial-order relations and provide a theoretically structured representation of hierarchical inclusion [32]. Simpler Poincaré-space objectives based on attraction between related prototypes and separation of unrelated categories do not impose the same guarantees. Such objectives can encourage embeddings to reflect taxonomy-dependent proximity, but they do not necessarily enforce radial depth ordering, entailment-cone constraints, or an exact order-preserving embedding. They should therefore be interpreted as taxonomy-aware regularization in hyperbolic space rather than as explicit hyperbolic order embedding.

Overall, the literature reveals three complementary requirements for fine-grained oriented ship detection: accurate orientation-aware localization, computationally efficient contextual and cross-level feature interaction, and semantic representation learning that respects the known ship taxonomy. Existing oriented detectors primarily address geometric localization; contextual remote-sensing architectures improve receptive-field adaptation and feature fusion; and hierarchical or hyperbolic methods provide mechanisms for representing semantic relationships. However, integrating these directions within a dense end-to-end oriented detector remains comparatively underexplored. The proposed YOLO26-OBB-HMS framework addresses this intersection by combining pre-FPN/PAN directional context, computationally bounded cross-level Feature Guidance, assignment-aligned multi-level supervision, and training-time Poincaré taxonomy objectives while retaining the native YOLO oriented-detection pathway for final inference. Accordingly, the contribution lies not in treating directional convolution, windowed attention, hierarchical supervision, or hyperbolic geometry as individually novel concepts, but in adapting and integrating them for hierarchy-aware dense oriented ship detection.

## 3. Methodology

### 3.1. Problem Formulation and Taxonomy Encoding

We consider fine-grained oriented ship detection in optical remote-sensing imagery. Given an image, the detector predicts oriented bounding boxes together with native leaf-class scores. Each box is represented by its center coordinates, side lengths, and orientation angle. The semantic vocabulary is encoded by a fixed taxonomy in which every retained leaf has exactly one intermediate and one coarse ancestor. Let *K*, *M*, and *C* denote the numbers of retained leaf, intermediate, and coarse categories, respectively; *d* denotes the semantic embedding dimension. The deterministic ancestor mappings are

*y^m^* = *M*(*y*),  *y^c^* = *C*(*y*),  *y* ∈ {1, …, *K*}. (1)

Hierarchical supervision assigns one categorical target at each semantic level; it does not imply multiple simultaneously positive leaf classes for a ship. In the retained HRSC2016 vocabulary, (*C*, *M*, *K*) = (1, 4, 21). The single coarse logit is interpreted as Ship versus background because a one-class softmax objective would be degenerate. In the retained ShipRSImageNet vocabulary, (*C*, *M*, *K*) = (2, 17, 37); the universal Ship root is implicit in foreground assignment. These counts refer to the prepared experimental vocabularies rather than the complete original dataset taxonomies [25,26].

YOLO-OBB annotations provide the leaf identifier and normalized quadrilateral geometry. During loss computation, the leaf label returned by the host assigner is mapped to its intermediate and coarse ancestors through fixed lookup tensors, so parent targets cannot be independently relabeled. The training launcher also invokes the repository’s OBB-export audit before training when data auditing is enabled. Geometry conversion and source-annotation validation are preprocessing responsibilities; they are distinct from semantic consistency among the model’s predicted hierarchy levels.

### 3.2. Architecture and Feature Routing

The proposed framework, termed YOLO26-OBB-HMS, extends the end-to-end YOLO26-OBB implementation. The active architecture consists of ACB, local Feature Guidance (FG), parent-level auxiliary classifiers, Poincaré auxiliary classification, and taxonomy regularization. The host backbone and FPN/PAN neck supply three dense detection maps at strides 8, 16, and 32:

(2) $$\{P_{3},P_{4},P_{5}\}=N\left(C_{3},C_{4},C_{5}\right),\;\;P_{l}\in R^{B\times c_{l}\times h_{l}\times w_{l}},\;l\in \{3,4,5\}.$$

Here, *B* is the mini-batch size, *c_l_* is the channel dimension, and *h_l_* and *w_l_* are the spatial dimensions; the notation *C_l_* for backbone lateral features is distinct from *C*, the number of coarse classes. Equation (2) gives the native neck inputs; for the ACB-enabled variant, the selected lateral inputs become the enriched features of Equation (5). The custom model intercepts backbone layers 4, 6, and 10, corresponding to the stride-8, stride-16, and stride-32 lateral features used by the YOLO26 FPN/PAN. At strides 8 and 16, the original activation continues through the deeper sequential backbone while the ACB-enriched copy is saved for the later lateral connection. At stride 32, the enriched output also replaces the sequential feature entering the neck. ACB therefore modifies the features supplied to pyramid fusion without recursively replacing all intermediate backbone activations.

**Figure 1:**
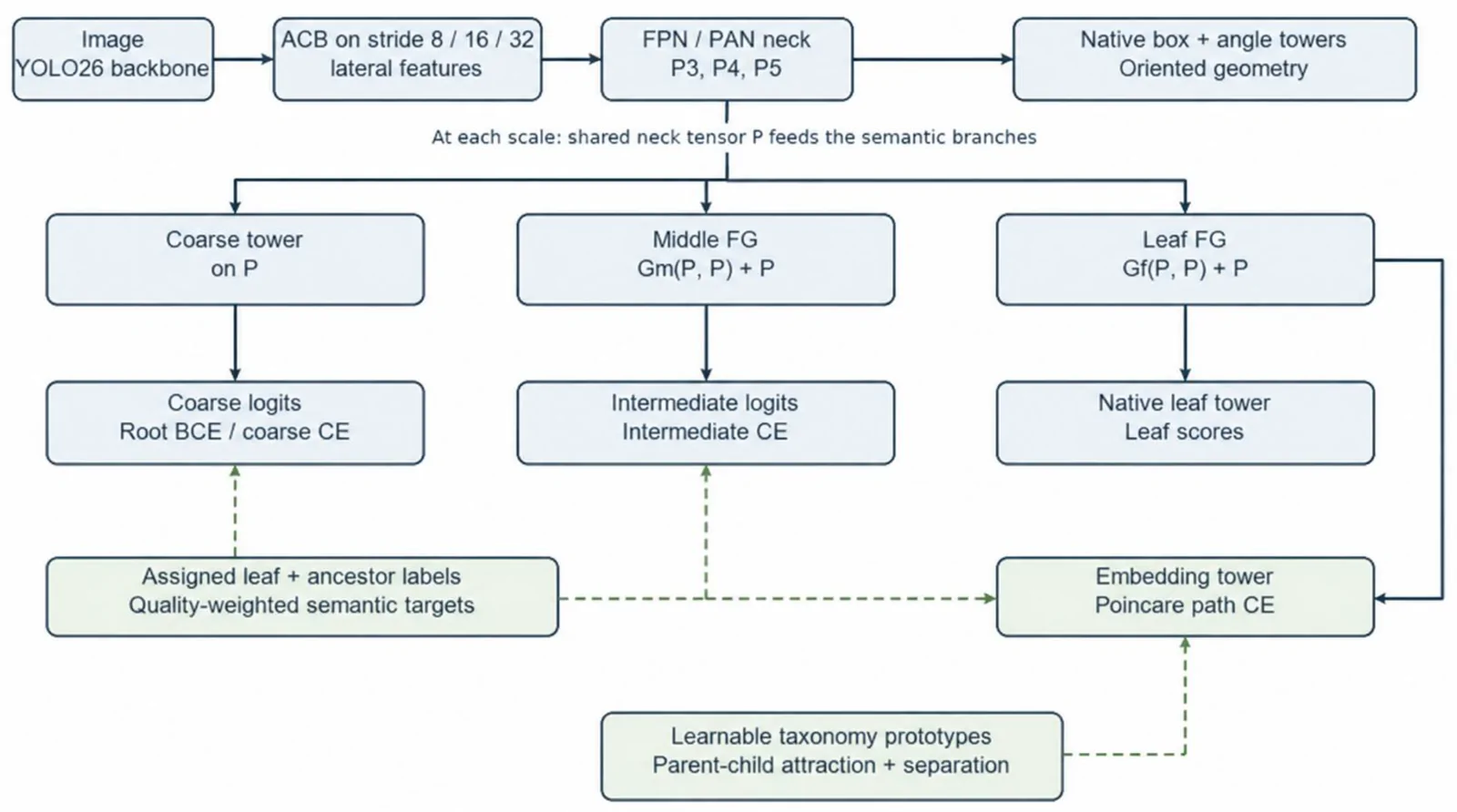
Architecture of YOLO26-OBB-HMS. ACB enriches backbone features, FG refines hierarchical representations, and Poincaré supervision is used only during training.

At each neck scale, the native box-distance and angle towers operate directly on *P_l_*. The native leaf-classification tower receives the FG-refined leaf representation. Separate auxiliary towers produce coarse logits, intermediate logits, and a 64-dimensional embedding by default. Figure 1 summarizes the proposed YOLO26-OBB-HMS data flow.

### 3.3. Axis-Wise Context Block Before Pyramid Fusion

For each selected backbone lateral feature *C_l_*, ACB computes complementary horizontal and vertical depthwise responses. With the launcher default at *k* = 7 and batch normalization BN, the responses are

*H_l_* = *BN*(*DWConv*_1×*k*_(*C_l_*)),*V_l_* = *BN*(*DWConv_k_*_×1_(*C_l_*)). (3)

Global average pooling (GAP) supplies an image- and scale-dependent descriptor to a two-layer 1 × 1 gating network. The hidden layer uses one quarter of the backbone lateral feature channels, which are rounded down with a minimum width of one channel. SiLU follows the first projection, and the second produces two directional logits. Softmax over those logits yields the mixture coefficients:

(4) $$(a_{l}^{h},a_{l}^{v})=\mathrm{softmax}\left(W_{2}\mathrm{SiLU}(W_{1}\mathrm{GAP}(C_{l}))\right).$$

For a given image and feature scale, the two mixture coefficients are broadcast across all channels and spatial locations. The gated directional response is projected by a 1 × 1 convolution, batch-normalized, added to the input through a residual connection, and passed through SiLU:

(5) $${\overset{\sim }{C}}_{l}=\mathrm{SiLU}\left(C_{l}+\mathrm{BN}\left(W_{p}(a_{l}^{h}H_{l}+a_{l}^{v}V_{l})\right)\right).$$

ACB therefore introduces anisotropic context before multi-scale fusion. Its axes remain those of the feature map: it neither estimates a canonical vessel axis nor guarantees rotational equivariance. Because the enriched lateral features are subsequently consumed by the shared FPN/PAN, ACB can indirectly influence leaf classification, box regression, and angle estimation.

### 3.4. Local Feature Guidance and Semantic Prediction

At each neck scale, the coarse, intermediate, and leaf semantic branches share the same raw tensor *P*_l_. Let $U_{l}^{c}$, $U_{l}^{m}$, and $U_{l}^{f}$ denote these branch inputs. The semantic routing is

(6) $$\begin{array}{c} U_{l}^{c}=U_{l}^{m}=U_{l}^{f}=P_{l},\\ F_{l}^{f}=U_{l}^{f}+G_{l}^{f}\left(\;U_{l}^{m},U_{l}^{f}\right),\\ F_{l}^{m}=U_{l}^{m}+G_{l}^{m}\left(\;U_{l}^{c},U_{l}^{m}\right). \end{array}$$

The intermediate representation adds guidance computed from the shared coarse/raw input, while the leaf representation adds guidance computed from the shared raw intermediate input. Crucially, the leaf FG module receives $U_{l}^{m}$ rather than the already refined $F_{l}^{m}$. Although both FG calls start from the same underlying *P_l_* tensor, they are not computationally shared: each has independent MultiheadAttention, LayerNorm, projection, and feed-forward parameters. Consequently, input sharing avoids a separate raw feature re-extraction path but does not remove the cost of cross-attention. With two FG calls at each of the three pyramid scales, the final semantic head executes six local FG modules per forward pass. Their attention-score term scales as *O*(*h_l_n_l_c_l_*) for fixed window area n, while the projection and feed-forward terms scale as $O(h_{l}n_{l}{c_{l}}^{2})$; this duplicated channel-mixing work is a principal contributor to the additional FLOPs and latency quantified in Section 4.7.

For upper and lower inputs U and V, each FG module pads the bottom and right edges as needed, partitions the feature map into non-overlapping 7 × 7 windows, and processes *n* = 49 tokens per window. The lower input supplies the queries, and the upper input supplies the keys and values. Let *X_U_* and *X_V_* be the window-token matrices and *d_h_* be the channel dimension of one attention head. For head *j*, omitting affine biases for clarity,

(7) $$A_{j}=\mathrm{softmax}\left(\frac{Q_{j}K_{j}^{T}}{\sqrt{d_{h}}}\right)V_{j},Q_{j}=X_{V}W_{j}^{Q},K_{j}=X_{U}W_{j}^{K},V_{j}=X_{U}W_{j}^{V}.$$

PyTorch (v2.13.0+cu130) MultiheadAttention performs the head concatenation and output projection. The projected attention response is then layer-normalized and transformed by a two-layer feed-forward network with hidden width 4*c_l_* and GELU activation. The caller adds this FG output residually to the corresponding raw lower-level feature, as expressed by Equation (6). Denoting GELU by *φ* and inverse window reconstruction by Unwin,

*G*(*U*,*V*) = *Unwin*(*W_b_ϕ*(*W_a_LN*(*Concat_j_*(*A_j_*)*W^O^*))). (8)

Inverse window reconstruction restores the spatial feature map, and bottom/right padding is cropped away. The implementation uses neither shifted windows nor an explicit mask for padded tokens; consequently, boundary windows may include padded key/value positions. The attention-head count is the largest divisor of *c_l_* not exceeding eight. For a fixed window area n, attention-score computation scales as *O*(*h_l_w_l_c_l_*), linearly with the number of spatial locations, whereas global attention scales quadratically. Learned projections and feed-forward layers retain a channel-mixing cost of $O(h_{l}w_{l}c_{l}^{2})$.

The coarse auxiliary classifier consumes the raw neck feature *P_l_*, the intermediate auxiliary classifier consumes the refined intermediate representation, and the native leaf tower consumes the refined leaf representation. A separate tower maps the refined leaf representation $F_{l}^{f}$ to a 64-dimensional visual embedding for hyperbolic supervision, consistent with Figure 1. Each auxiliary tower comprises a depthwise 3 × 3 convolution with batch normalization and SiLU, a pointwise Conv block, and a final 1 × 1 projection. Native leaf logits retain sigmoid scoring. Multi-class parent logits use softmax cross-entropy during training and do not replace native leaf confidences.

### 3.5. Assignment-Aligned Hierarchical Supervision

For either end-to-end prediction branch, let *A* denote all dense locations in the mini-batch and *F* ⊆ *A* the locations selected as foreground by the host rotated Task-Aligned Assigner. The custom auxiliary loss recomputes the same assignment form from detached native leaf scores and detached decoded boxes, then obtains the matched ground-truth leaf index and the host quality-aware soft target vector. It also applies the same preprocessing filter used by the custom criterion, removing training boxes whose resized width or height is below two pixels. Let *t_ik_* be the detached native soft target for class *k* at location *i*. Define the assignment quality weight and normalization as

(9) $$q_{i}=\sum_{k=1}^{K}t_{\mathrm{ik}},Z=\max\left(1,\sum_{i\in F}q_{i}\right).$$

For intermediate logits $a_{i}^{m}$ and, when *C* > 1, coarse logits $a_{i}^{c}$, the parent targets are deterministic mappings of the matched leaf label. Writing *π^m^* = *softmax*(*a^m^*) and *π^c^* = *softmax*(*a^c^*) their categorical cross-entropy losses are evaluated only at assigned foreground locations and are weighted by the detached assignment quality *q_i_*:

(10) $$L_{m}=-\frac{1}{Z}\sum_{i\in F}q_{i}\log\pi_{i,M(y_{i})}^{m}\;L_{c}=-\frac{1}{Z}\sum_{i\in F}q_{i}\log\pi_{i,C(y_{i})}^{c},C>1.$$

Equation (10) applies to the coarse level only when *C* > 1. For HRSC2016, a single-logit softmax cross-entropy would be identically zero. The implementation therefore trains the single coarse logit with binary cross-entropy over all dense locations, using the sum of the native soft class targets (clipped to one) as a quality-aware Ship-versus-background target:

(11) $$L_{c}=\frac{1}{Z}\sum_{i\in A}\mathrm{BCEWithLogits}(a_{i}^{c},r_{i}),r_{i}=\min\left(1,\sum_{k=1}^{K}t_{\mathrm{ik}}\right).$$

This root objective models Ship versus background rather than discrimination among ship groups. In the custom loss, both parent losses are set to zero when a branch contains no assigned foreground locations; consequently, the dense HRSC root BCE is evaluated only for branch calls that contain at least one positive assignment. The taxonomy lookup tensors and quality weights are treated as fixed targets, and gradients do not pass through the assignment computation. The same normalization *Z* defined in Equation (9) is used in both Equations (10) and (11). The sums in Equation (10) are restricted to *i* ∈ *F* because the multi-class parent cross-entropy terms are defined only on assigned foreground locations, whereas Equation (11) sums over *i* ∈ *A* because the binary root objective explicitly includes background locations. Thus, the difference in index domains is intentional and does not represent a normalization mismatch. The same two-pixel threshold is also applied to the evaluation ground truth after resizing to the evaluation input resolution

### 3.6. Poincaré Path Supervision

The hyperbolic module connects the FG-refined visual embeddings to learnable taxonomy prototypes during training. Let *κ* > 0 denote the curvature magnitude, so the manifold curvature is −*κ*. Visual embeddings and learnable tangent-space prototype vectors are mapped from the origin into the Poincaré ball by

(12) $$B_{\kappa }^{d}=\{x\in R^{d}:\kappa \|x{\|}^{2}<1\},\exp_{0}^{\kappa }(v)=\frac{\tanh(\sqrt{\kappa }\|v\|)}{\sqrt{\kappa }\|v\|}v.$$

The mapping is well defined at *v* = 0 through the implementation’s norm clamp. Separate trainable tangent vectors are maintained for every retained leaf and intermediate class, and for coarse classes only when C > 1. The same exponential map is applied to visual embeddings and prototypes. Their geodesic distance is

(13) $$d_{\kappa }(x,y)=\frac{1}{\sqrt{\kappa }}\mathrm{arcosh}\left(1+\frac{2\kappa \|x-y{\|}^{2}}{\left(1-\kappa \|x{\|}^{2})(1-\kappa \|y{\|}^{2}\right)}\right).$$

For a candidate leaf *k*, let P(k) denote its informative taxonomy path. HRSC2016 uses the leaf and intermediate prototypes because its single Ship root is excluded from hyperbolic geometry; ShipRSImageNet additionally includes the two-class coarse ancestor. The separate HRSC coarse logit remains supervised by Equation (11). With temperature *τ* > 0 and mapped visual embedding *x_i_*, the path score and auxiliary leaf-classification loss are

(14) $$s_{\mathrm{ik}}=-\frac{1}{\tau \left|P\left(k\right)\right|}\sum_{p\in P(k)}d_{k}(x_{i},p),\;L_{h}=-\frac{1}{Z}\sum_{i\in F}q_{i}\log\left(\frac{\exp\left(s_{i,y_{i}}\right)}{\sum_{k=1}^{K}\exp\left(s_{\mathrm{ik}}\right)}\right).$$

Equation (14) averages the geodesic distance over the active path prototypes and scales the resulting negative distance by temperature τ. Its softmax cross-entropy compares each assigned visual embedding with every retained leaf candidate. The path score is an auxiliary training quantity and is not used for inference ranking.

Hyperbolic calculations are explicitly performed in float32 with autocasting disabled. Norms and Poincaré denominators are clamped by *ε* = 10^−5^, mapped points are projected inside radius $(1-\varepsilon )/\sqrt{\kappa }$, and the arcosh argument is lower-bounded by 1 + *ε*. Prototype tangent vectors are initialized from N(0, 0.01^2^). These operations are numerical safeguards rather than additional hierarchy constraints. When *F* is empty, the visual hyperbolic classification term is zero, but prototype-only taxonomy regularization remains active.

### 3.7. Taxonomy Regularization

Let $p_{k}^{f}$, $p_{j}^{m}$, and $p_{r}^{c}$ denote embedded leaf, intermediate, and coarse prototypes, and let *C_m_*(*j*) be the unique coarse parent of intermediate class *j*. Direct parent–child attraction is averaged separately at each active edge level:

(15) $$L_{\mathrm{att}}=\frac{1}{K}\sum_{k=1}^{K}d_{\kappa }\left(p_{k}^{f},p_{M\left(k\right)}^{m}\right)+\frac{1_{C>1}}{M}\sum_{j=1}^{M}d_{\kappa }\left(p_{j}^{m},p_{C_{m}\left(j\right)}^{c}\right).$$

To discourage prototype collapse, define *R* as all unordered pairs of distinct active prototypes except direct parent–child pairs. The separation term and total taxonomy objective are

(16) $$L_{\mathrm{sep}}=(1/\left|R\right|)\sum_{(u,v)\in R}[\mu -d_{\kappa }(p_{u},p_{v}){]}_{+},\;\;\;L_{\mathrm{tax}}=L_{\mathrm{att}}+L_{\mathrm{sep}}.$$

Here, μ > 0 is the separation margin and [.]_+_ denotes the positive part. The implementation constructs all unordered pairs among active coarse, intermediate, and leaf prototypes, then removes only direct parent–child edges from the separation mask. Thus, a non-adjacent ancestor–descendant pair (for example, a coarse prototype and a leaf below one of its intermediate children) is still subject to margin separation. The resulting objective encourages local parent–child proximity and broader prototype separation, but it does not impose entailment cones, radial depth ordering, or a guaranteed embedding of the full ancestor partial order [32]. The taxonomy term is independent of foreground presence and remains active even when the visual hyperbolic Cross Entropy is zero.

### 3.8. Joint Objective and Gradient Flow

The custom criterion delegates to the host v8OBBLoss and appends the hierarchical losses. The native box, leaf-classification, distance-regression, and angle objectives therefore follow Ultralytics (v8.4.12). With reg_max = 1, the distance term is normalized L1 regression, logged as l1_loss; the configuration key dfl controls its gain even though no multi-bin distribution focal loss is used. For each end-to-end branch, the native objective is summarized as

ℒ_native_ = *λ*_box_ℒ_pIoU_ + *λ*_cls_ℒ_leaf_ + *λ*_reg_ℒ_L1_ + *λ*_ang_ℒ_angle_. (17)

The probabilistic rotated-overlap term is evaluated on assigned positives, the L1 term compares four encoded box distances, the native leaf objective uses sigmoid BCE over dense soft targets, and the angle objective accounts for the π-periodicity of oriented rectangles. The archived HRSC2016 configurations set λ*_box_* = 7.5, λ*_cls_* = 0.5, λ*_reg_* = 1.5 and λ*_ang_* = 1.0. These are host-detector objectives and gains, not newly proposed losses.

The branch objective adds the hierarchical, hyperbolic, and taxonomy losses:

𝒥*_b_* = ℒ_native,*b*_ + *λ_m_*ℒ*_m_*_,*b*_ + *λ_c_*ℒ*_c_*_,*b*_ + *λ_h_*ℒ*_h_*_,*b*_ + *λ*_tax_ℒ_tax,*b*_. (18)

In the final YOLO26-OBB-HMS objective, hyperbolic cross-entropy and taxonomy regularization are enabled in addition to the parent-level auxiliary losses; no cross-level probability-consistency loss is added. With the launcher defaults, the intermediate, coarse, and hyperbolic gains are 1.0, the taxonomy gain is 0.05, and *κ* = *τ* = 1.0. These hyperparameters remain configurable; the values used in the reported experiments correspond to the operating point selected by the local screening described in Section 4.2 and are interpreted as the best-performing setting within the examined ranges rather than as a unique theoretical or globally optimal solution.

YOLO26 end-to-end training maintains separate one-to-many and one-to-one prediction towers. The custom head deep-copies the FG and auxiliary semantic modules for the one-to-one branch, and both branch criteria receive the same types of hierarchical supervision with branch-specific assignments. In the referenced host criterion, the one-to-many assigner uses top − k = 10 and the one-to-one path uses top − k = 7 followed by a secondary top − *k*_2_ = 1. The combined scalar objective follows the host end-to-end weighting schedule:

ℒ_total_ = *B*[*ω*(*t*)𝒥_many_ + (1 − *ω*(*t*))𝒥_one_.] (19)

Let t denote the branch-weight schedule step and *T* denote its configured horizon. The branch weight is *w*(*t*) = 0.1 + 0.7 max(1 − *t*/max(*T* − 1, 1), 0); for *T* > 1, it decreases from 0.8 to 0.1 by *t* = *T* − 1. Both branch criteria share the HyperbolicTaxonomy module and its learnable prototypes, while branch-specific assignments determine their visual losses. The taxonomy term enters the total objective through the same branch weights.

During end-to-end training, the host one-to-one path receives detached feature inputs. Its losses therefore update the one-to-one prediction/semantic towers and the shared hyperbolic prototypes without propagating through those detached inputs into the neck, ACB, or backbone. The one-to-many branch supplies the feature-extractor gradients from native and auxiliary visual objectives. Within the selected YOLO26-OBB-HMS configuration, the intermediate classifier uses $F_{l}^{m}$, while the native leaf classifier and embedding tower use $F_{l}^{f}$; because leaf FG attends to the raw intermediate input rather than $F_{l}^{m}$, there is no recursive gradient path from the leaf FG computation through the intermediate FG output.

## 4. Experimental Setup and Evaluation Protocols

### 4.1. Datasets and Evaluation Protocols

HRSC2016 [25] comprises 1061 images containing 2976 annotated ship instances, distributed across the official training, validation, and test partitions with 436, 181, and 444 images, respectively. Following the typed-ship categorization adopted in HMS-Net [9], the prepared HRSC2016 task retains 21 leaf-level ship classes organized into four intermediate groups under a generic Ship root category. The official training and validation partitions are combined to form the development pool. From this pool, a fixed internal subset is reserved exclusively for architecture and checkpoint selection, while the remaining images are used for model fitting. After all architectural and checkpoint-selection decisions are finalized, performance is assessed on the filtered official test partition. Prior to the internal development split, the retained development pool contains 570 images and 1308 annotated objects, whereas the filtered test partition contains 404 images and 963 objects.

ShipRSImageNet [26] originally contains 3435 remote-sensing images covering a fine-grained ship taxonomy. In the present study, the original official training, validation, and test partitions were not used. Instead, the dataset was re-prepared according to the retained class vocabulary employed in our experiments. Specifically, the Dock category was excluded, together with its associated images, resulting in a prepared set of 3121 images. The remaining images were repartitioned independently of the official dataset split into 2198 training, 373 validation, and 550 final-evaluation images. The retained semantic vocabulary comprises 37 typed ship classes organized into 17 intermediate groups and two coarse-level domains. All training, model-selection, and quantitative evaluation procedures reported for ShipRSImageNet were conducted exclusively on these newly prepared partitions rather than on the dataset’s original official train, validation, and test partitions.

The constructed training partition comprises 4458 retained ship instances across 2198 images, whereas the validation partition consists of 1091 retained annotation entries in 373 images. Furthermore, the final-evaluation partition encompasses 1404 annotation entries across 550 images, of which 1403 unique instances are retained by the Ultralytics loader.

**Figure 2:**
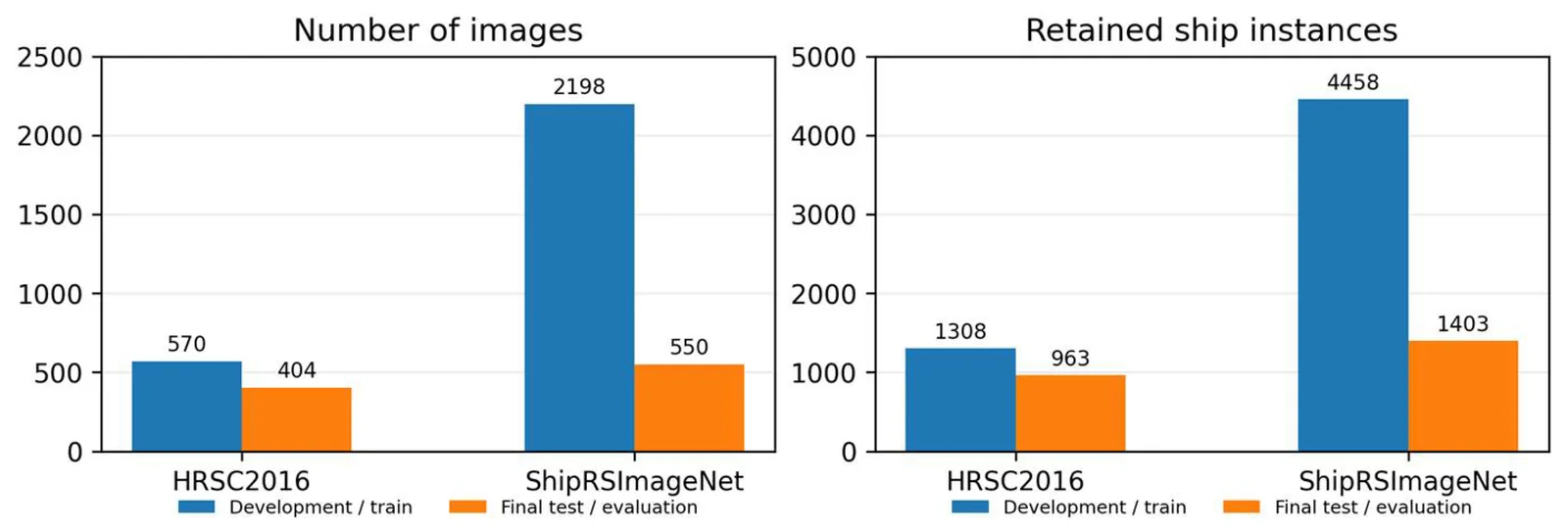
Image and retained instance counts for the prepared HRSC2016 and ShipRSImageNet partitions.

Figure 2 illustrates the source partition structures established for both datasets. For ShipRSImageNet, the final fitting and internal-validation allocation is described above, whereas the HRSC2016 development bar represents the combined development pool prior to its internal model-selection split.

### 4.2. Implementation and Training Settings

YOLO-based experiments were implemented in PyTorch using the Ultralytics (v8.4.12) YOLO26 oriented-object-detection framework. The native scale-specific detectors are denoted as YOLO26n-OBB and YOLO26s-OBB, whereas their hierarchy-aware extensions are referred to as YOLO26n-OBB-HMS and YOLO26s-OBB-HMS. Unless otherwise stated, all YOLO26-OBB and YOLO26-OBB-HMS experiments used the same training configuration: pretrained YOLO26-OBB weights, a maximum training budget of 200 epochs, a batch size of 16, four data-loader workers, and a nominal input size of 640 pixels. The optimizer = auto option resolved to AdamW in the documented runs, with an initial learning rate of 0.0004 and beta coefficients of (0.9, 0.999). Consequently, the configured lr0 = 0.01 and momentum = 0.937 values were superseded by the automatically selected optimizer settings. Weight decay was set to 0.0005 for decay-eligible parameters and to zero for normalization and bias parameters. Gradient accumulation over four optimization steps resulted in an effective batch size of 64. Automatic mixed precision (AMP) and deterministic execution were enabled. Following a three-epoch warm-up period, the learning rate was linearly decayed to 0.01 times its initial value.

During training, a size-based filter is applied to the rotated ground-truth boxes before foreground assignment. Boxes whose resized width or height is below two pixels are excluded from the training target set. The threshold is defined in the current network-input coordinate system after resizing. The detection criterion and the auxiliary hierarchical loss apply the same filter, ensuring that native detection and hierarchical supervision operate on consistent training targets. This filtering step is part of training-target preprocessing and is separate from the dataset-level class filtering described in Section 4.1.

Oriented R-CNN and RTMDet-R-s [33] were implemented using MMRotate-compatible pipelines [34] and were included as cross-framework reference methods, while HMS-Net was implemented following its original methodological formulation. RTMDet-R-s and HMS-Net were evaluated with an input resolution of 640 × 640 pixels to provide a consistent nominal image size with the YOLO-based experiments. Their remaining architecture-specific training, optimization, assignment, augmentation, and post-processing procedures followed the corresponding method-specific configurations. Oriented R-CNN likewise retained its own recommended implementation settings and input preprocessing rather than being forced to adopt the YOLO training recipe. Thus, the comparative experiments standardized the evaluation protocol and, where explicitly stated, the nominal input resolution, but did not impose a common optimization strategy across fundamentally different detector architectures.

To ensure a consistent cross-method comparison, predictions generated by Oriented R-CNN, RTMDet-R-s, HMS-Net, YOLO26-OBB, and YOLO26-OBB-HMS were converted into a unified output representation and independently evaluated using the same COCO-style AP definitions on identical prepared dataset partitions and retained class vocabularies. Accordingly, the reported cross-framework results reflect a common evaluation protocol despite differences in the native training and inference procedures of the individual methods.

For the HRSC2016 ablation study, each Y0 and H0–H15 configuration was trained in five independent runs using the common YOLO training configuration described above, the same dataset split, and an identical checkpoint-selection procedure. The run-to-seed mapping is provided in Section 4.3, while the individual measurements and their cross-configuration pairing are reported in Supplementary Table S3. For each run, checkpoint selection was performed exclusively on the fixed internal selection subset using the Ultralytics fitness criterion based on mAP50:95. Architectural comparisons, including the selection of the final H7 configuration, were likewise conducted solely on this internal subset. Once the architecture and checkpoint were fixed, the retained model was evaluated on the filtered official HRSC2016 test partition. Consequently, test-set metrics were not used for either architecture selection or checkpoint-epoch selection.

The hierarchy-aware YOLO26-OBB-HMS configurations additionally shared a common set of module-specific hyperparameters. The Axis-wise Context Block (ACB) used a kernel length of 7, Feature Guidance (FG) operated with 7 × 7 local windows, and the semantic embedding dimension was fixed at 64. For configurations employing Poincaré supervision, the hyperbolic classification loss gain was set to 1.0 and the taxonomy-regularization gain to 0.05. The Poincaré operating point was selected through a local one-factor-at-a-time screening on the development/model-selection data before the final ablation comparisons. Holding the remainder of H7 fixed, curvature was evaluated at κ ∈ {0.1, 1.0, 10.0}, temperature at τ ∈ {0.5, 1.0, 2.0}, and the taxonomy-regularization gain at λ*_tax_* ∈ {0.01, 0.05, 0.20}, with the other two quantities fixed at their respective reference values in each sweep. The setting κ, τ, λ*_tax_* = (1.0, 1.0, 0.05) provided the most favorable combination of selection-subset performance and training stability and was therefore retained for the subsequent Poincaré-related configurations, where applicable. These screening trials were used to select a common operating point rather than as part of the five-seed component-ablation study; consequently, they are not represented as additional H configurations or included in the statistical contrast family. The selected setting is regarded as the best-performing operating point under the examined one-factor-at-a-time screening, rather than as a unique or globally optimal solution. These hyperbolic objectives were used exclusively as auxiliary training signals and were not fused with the native YOLO leaf-class scores during inference. Within the ablation design, H9 preserves the ACB, FG, and parent-supervision components of H7 while keeping MRFR and proxy learning disabled; however, both Poincaré cross-entropy and taxonomy regularization are removed, and the HyperbolicTaxonomy module is therefore not instantiated.

### 4.3. Ablation Design

Table 1 defines the HRSC2016 n-scale ablation matrix, with native YOLO26n-OBB (Y0) as the reference. H0–H5 examine feature modules, parent supervision, and proxy learning without a path objective. H5/H6 compares proxy learning with Poincaré path cross-entropy plus taxonomy regularization; this replacement changes both supervision and geometry. H7/H6 and H8/H6 examine ACB and MRFR, respectively, on the otherwise matched H6 background.

With H7 as the reference, H9 disables both Poincaré terms, H11 retains path cross-entropy alone, and H12 retains taxonomy regularization alone. H7/H10/H14/H15 form a 2 × 2 FG-by-parent-supervision design with ACB and Poincaré supervision held fixed. H13 matches H7 in detector, feature modules, parent targets, embedding dimension, taxonomy paths, prototypes, temperature, margin, and loss weights; only the Poincaré map and geodesic distance are replaced by direct Euclidean representations and L2 distance. H10 disables FG while retaining H7’s other listed settings, making H7/H10 the matched FG-on/FG-off comparison with parent supervision active. Runs 1–5 correspond to random seeds 0, 42, 100, 120, and 150, respectively; corresponding runs across configurations were paired for analysis.

The local κ, τ, and λ*_tax_* screening described in Section 4.2 was used only to establish the common Poincaré operating point and is therefore not represented as additional H configurations in Table 1; the formal component ablations below use the selected values without per-configuration retuning.

### 4.4. Ablation Results and Discussion

Table 2 summarizes the results obtained from five independent HRSC2016 runs for each configuration, reporting the mean ± sample standard deviation together with the observed minimum–maximum range. Ten targeted, seed-matched contrasts were evaluated for both mAP50 and mAP50:95 using two-sided paired *t*-tests, and Holm’s procedure was applied jointly to the resulting family of 20 *p*-values.

Relative to the native Y0 baseline, the complete H7 configuration increased the mean mAP50 from 73.07 ± 0.07% to 77.42 ± 0.18% and the mean mAP50:95 from 66.14 ± 0.22% to 67.46 ± 0.33%, corresponding to absolute gains of 4.35 and 1.32 percentage points (pp), respectively. H7 outperformed Y0 on both metrics in all five matched runs. In addition, the observed mAP50:95 ranges did not overlap (H7: 67.11–67.96%; Y0: 65.85–66.38%), and the Holm-adjusted paired *p*-values were 4.80 × 10^−6^ for mAP50 and 0.0412 for mAP50:95. These results provide consistent evidence that the complete hierarchy-aware configuration improves aggregate performance under the evaluated HRSC2016 protocol, although this comparison alone does not identify the contribution of any individual component.

The remaining ablations provide more specific insight into the behavior of the proposed formulation. All five proxy-based configurations (H0, H1, H2, H4, and H5) achieved lower mean scores than Y0 on both metrics. In the matched H1/H3 comparison, removing proxy learning while retaining ACB, MRFR, FG, and parent supervision increased mAP50 from 60.85% to 74.86% and mAP50:95 from 50.22% to 64.79%. Similarly, replacing the proxy-based formulation in H5 with the Poincaré-supervised formulation in H6 produced gains of 7.82 pp in mAP50 and 9.75 pp in mAP50:95. Because H5 and H6 differ in both supervision strategy and metric formulation, however, this contrast cannot be attributed specifically to hyperbolic geometry. Rather, these results indicate that the choice of hierarchical training objective has a substantial influence on the behavior of the detector.

The architectural controls further support the selection of H7. Adding ACB to the otherwise matched H6 configuration increased mean mAP50 and mAP50:95 by 1.40 and 1.38 pp, respectively, with non-overlapping five-run ranges and Holm-adjusted paired *p*-values of 0.00356 and 0.00741. Nevertheless, the opposite trend observed in the proxy-based H0/H1 comparison indicates that the effect of ACB depends on the surrounding training formulation and should not be interpreted as configuration-independent. By comparison, adding MRFR to H6 increased mAP50 by 0.59 pp, while mAP50:95 changed by only 0.01 pp. Among the evaluated alternatives, H7 therefore combines a favorable ACB contrast with stronger performance on both reported metrics than the MRFR-based H8 configuration.

The path-loss controls reveal a similarly metric-dependent pattern. Relative to H9, which removes both Poincaré terms, H7 improved mAP50 by 0.14 pp and mAP50:95 by 0.83 pp, with corresponding Holm-adjusted *p*-values of 0.618 and 0.0412. H7 also outperformed the taxonomy-only H12 configuration by 3.25 pp in mAP50 and 1.79 pp in mAP50:95 (adjusted *p* = 0.000901 and 0.00814, respectively). In contrast, the differences between H7 and the path-cross-entropy-only H11 configuration were comparatively small (+0.06 pp and +0.53 pp), with adjusted *p*-values of 0.618 and 0.111. Taken together, these comparisons provide the clearest support for the complete path formulation relative to H12 and, for mAP50:95, relative to H9. They do not, however, establish an independent benefit of each individual loss term across both metrics.

The matched geometry control provides a more direct assessment of the Poincaré formulation. H13 retains the objectives and listed architectural settings of H7 while replacing the Poincaré mapping and geodesic distance with Euclidean representations and L2 distance. Under this matched comparison, H7 exceeded H13 by 1.73 pp in mAP50 and 1.63 pp in mAP50:95. The observed five-run ranges did not overlap, and the Holm-adjusted paired *p*-values were 0.00143 and 0.00274, respectively. These results favor the Poincaré formulation within the evaluated HRSC2016 setting, while not implying that hyperbolic geometry is universally superior across other datasets, architectures, or hyperparameter choices.

The FG and parent-supervision results indicate that these components should be interpreted jointly. When parent supervision is enabled, H7 exceeds the matched FG-disabled H10 configuration by 0.79 pp in mAP50 and 0.53 pp in mAP50:95. However, the Holm-adjusted *p*-values of 0.0502 and 0.0653 do not provide strong evidence for an isolated FG effect. Moreover, when parent supervision is disabled, the direction of the FG contrast reverses: H14, with FG enabled, underperforms the FG-disabled H15 configuration by 2.88 pp in mAP50 and 2.80 pp in mAP50:95. Parent supervision likewise exhibits a favorable contrast when FG is active (H7 versus H14: +4.09/+2.81 pp), whereas its effect is mixed when FG is absent. These findings support the combined H7 formulation while indicating that neither FG nor parent supervision should be assigned a configuration-independent contribution.

Overall, H7 achieved the highest mean mAP50 and mAP50:95 among the evaluated configurations and provided the strongest joint performance profile in the ablation study. Nevertheless, several configurations approached H7 on individual metrics. H9 was only 0.14 pp lower in mAP50, although it remained 0.83 pp lower in mAP50:95, while H15 achieved an almost identical mAP50:95 (67.45% versus 67.46%) but remained 1.21 pp lower in mAP50. These near ties should therefore not be interpreted as strict rankings. Rather, the five-run comparison with the native baseline, together with the matched ACB and Euclidean controls, provides the clearest empirical support for H7, whereas the remaining contrasts define the limits of the component-level conclusions that can be drawn from the present experiments.

### 4.5. Class-Wise Comparison with Oriented Detection Baselines

Class-wise AP50 was used as the primary diagnostic metric to examine how the hierarchy-aware formulation affects individual fine-grained ship categories, thereby revealing class-specific behavior that may be obscured by aggregate performance measures. AP50 was computed at an intersection-over-union threshold of 0.50 using a common COCO-style evaluation pipeline applied identically to all detectors. Framework-native metric summaries from Ultralytics and MMRotate were therefore excluded from this comparison. The final row of Table 3 reports mAP50 over the 21 retained HRSC2016 leaf classes.

Table 3 compares the native YOLO26-OBB detectors and their hierarchy-aware counterparts with Oriented R-CNN, RTMDet-R-s, and HMS-Net on the filtered HRSC2016 test partition. All predictions were evaluated using the same retained class vocabulary and common AP50 definition. Although this standardization ensures comparability with respect to evaluation data and metric computation, the external detector families retain their architecture-specific training, optimization, assignment, and implementation procedures. Accordingly, the scale-matched YOLO26-OBB versus YOLO26-OBB-HMS comparisons provide the most controlled evidence for the effect of the proposed hierarchy-aware formulation, whereas the external methods provide broader cross-framework performance context.

As shown in Table 3, the hierarchy-aware formulation improves aggregate HRSC2016 performance at both model scales, although the magnitude and distribution of these gains differ across classes. At the n scale, YOLO26n-OBB-HMS achieves 77.5% mAP50, compared with 73.1% for the scale-matched native YOLO26n-OBB, corresponding to an absolute improvement of 4.4 percentage points (pp). Class-wise AP50 increases in 14 of the 21 retained categories, remains unchanged in one, and decreases in six. The largest improvements occur for CRU (+28.0 pp), OXO (+17.2 pp), MS (+13.1 pp), CS (+12.9 pp), and PCF (+11.3 pp), whereas the largest reductions are observed for SATD (−12.3 pp), HVR (−8.7 pp), and AATD (−8.2 pp). The aggregate improvement therefore does not arise from a uniform upward shift across the taxonomy, but from a redistribution of class-specific detection performance.

The s-scale comparison exhibits a smaller but positive aggregate difference. YOLO26s-OBB-HMS increases mAP50 from 81.7% to 82.7%, yielding a gain of 1.0 pp over the native YOLO26s-OBB. The hierarchy-aware variant improves AP50 in 12 classes, remains unchanged in three, and decreases in six. Several categories show notable improvements, including SATD (81.9% → 89.8%), SUB (86.0% → 91.9%), OXO (76.8% → 82.7%), and TAS (93.1% → 96.6%). Conversely, reductions remain evident for classes such as CS2 (85.2% → 76.7%), AATD (79.2% → 73.1%), and WILD (76.0% → 71.5%). These class-level variations reinforce the observation that hierarchical supervision does not benefit all categories uniformly.

In the broader cross-framework comparison, YOLO26s-OBB-HMS attains the highest aggregate mAP50 in Table 3 at 82.7%, compared with 81.3% for Oriented R-CNN, 77.0% for HMS-Net, and 75.8% for RTMDet-R-s. The corresponding margins are +1.4, +5.7, and +6.9 pp, respectively. At the n scale, YOLO26n-OBB-HMS achieves 77.5%, exceeding HMS-Net by 0.5 pp and RTMDet-R-s by 1.7 pp, while remaining 3.8 pp below Oriented R-CNN. These cross-framework differences should be interpreted descriptively, however, because the compared methods retain distinct architectures and method-specific training and optimization pipelines. Consequently, the most controlled evidence remains the scale-matched comparison between each native YOLO26-OBB model and its corresponding YOLO26-OBB-HMS variant.

Taken together, the HRSC2016 results indicate that the proposed hierarchy-aware formulation provides a clear aggregate advantage over the native n-scale detector and a more modest improvement at the s scale. The stronger relative gain at the n scale suggests that hierarchical supervision and the associated contextual mechanisms may provide greater benefit when representational capacity is more constrained. However, because the present class-wise comparison does not independently isolate model capacity from the added hierarchy-aware components, this interpretation should be regarded as a plausible empirical observation rather than a demonstrated causal effect.

As shown in Table 4, the hierarchy-aware formulation also improves aggregate performance on the prepared ShipRSImageNet final-evaluation partition, although the gains are smaller and more class-dependent than those observed for the n-scale model on HRSC2016. At the n scale, YOLO26n-OBB-HMS achieves an mAP50 of 75.4%, compared with 74.1% for the native YOLO26n-OBB, corresponding to an absolute improvement of 1.3 percentage points (pp). Class-wise AP50 increases in 18 of the 37 retained categories, remains unchanged in four, and decreases in 15. The largest improvements are observed for YuDao LL (+26.3 pp), Asagiri DD (+21.4 pp), and Sanantonio AS (+16.9 pp), whereas the largest reductions occur for Medical Ship (−32.8 pp), YuTing LL (−12.4 pp), and YuZhao LL (−9.7 pp). These results indicate that the aggregate gain is driven by a substantial redistribution of class-specific performance rather than by a uniform improvement across the retained taxonomy.

At the s scale, YOLO26s-OBB-HMS achieves 80.6% mAP50, compared with 80.2% for YOLO26s-OBB, yielding a more modest gain of 0.4 pp. Performance improves in 22 classes, remains unchanged in one, and decreases in 14. The largest positive differences are observed for Test Ship (+14.9 pp), AOE (+12.2 pp), and Medical Ship (+8.0 pp), whereas Austin LL (−20.6 pp), Enterprise (−16.4 pp), and Tugboat (−10.4 pp) show the largest reductions. The direction and magnitude of these changes differ considerably across categories, further indicating that the hierarchy-aware components reshape fine-grained class discrimination rather than producing a uniform increase in AP50.

The scale-dependent class behavior is also noteworthy. Several categories that deteriorate at the n scale recover substantially at the s scale. For example, Medical Ship decreases from 86.6% to 53.8% in the n-scale comparison but increases from 71.5% to 79.5% at the s scale. Conversely, some categories that are relatively stable or competitive at the n scale deteriorate at the s scale, most notably Enterprise and Austin LL. This variation suggests that the effect of hierarchical supervision is influenced by model capacity and class-specific feature representation, although the present experiments do not isolate these factors sufficiently to support a causal interpretation.

In the broader cross-framework comparison, YOLO26s-OBB-HMS attains the highest aggregate mAP50 in Table 4 at 80.6%, exceeding YOLO26s-OBB (80.2%) by 0.4 pp and HMS-Net (78.3%) by 2.3 pp. By contrast, the n-scale hierarchy-aware model reaches 75.4%, which is 1.3 pp above its native counterpart but 2.9 pp below HMS-Net. Oriented R-CNN and RTMDet-R-s achieve 72.7% and 73.9%, respectively. These cross-framework differences should be interpreted descriptively because the detector families retain distinct architectures, optimization procedures, assignment mechanisms, and implementation pipelines. Accordingly, the scale-matched YOLO26-OBB versus YOLO26-OBB-HMS comparisons provide the most controlled evidence for the proposed formulation.

Overall, Table 4 indicates that the hierarchy-aware formulation provides a positive aggregate effect on ShipRSImageNet at both model scales, but the improvement is substantially smaller than the n-scale gain observed on HRSC2016 and is accompanied by pronounced class-specific gains and regressions. In particular, the 0.4-pp improvement of the s-scale model should be interpreted cautiously because seed-level dispersion is unavailable for these scale-specific checkpoint comparisons. The ShipRSImageNet results therefore support the usefulness of the proposed hierarchy-aware formulation at the aggregate level while also highlighting the need for broader multi-seed evaluation and further analysis of class-specific behavior.

### 4.6. Hierarchical Classification and Matched-Object Evaluation

The native leaf-level predictions and the directly predicted intermediate- and coarse-level outputs of YOLO26-OBB-HMS were evaluated on detections geometrically matched to ground-truth objects in the retained HRSC2016 and ShipRSImageNet evaluation partitions. This analysis was designed to assess semantic prediction quality conditional on successful localization and should therefore be distinguished from end-to-end class-aware detection AP. Within each model, the same set of selected detections and geometric matches was used across all semantic levels, enabling direct comparison of the leaf, intermediate, and coarse prediction heads on an identical cohort of localized objects.

For each dataset and model scale, the checkpoint selected according to the predefined model-selection protocol was evaluated in FP32 with a batch size of 1 and a nominal input size of 640 pixels. Aspect-ratio-preserving preprocessing was applied consistently throughout evaluation. For the head-level analysis, predictions from the native one-to-one detection branch were retained using a leaf-confidence threshold of 0.50, with a maximum of 300 detections per image.

Detection AP50 was computed using a class-aware, COCO-compatible oriented-object evaluation protocol. Within each image and class, detections were sorted in descending order of confidence and greedily matched one-to-one to the unmatched ground-truth oriented bounding box with the highest rotated polygon IoU, using an IoU threshold of 0.50. Successfully matched detections were counted as true positives, whereas unmatched detections and unmatched ground-truth objects contributed to false positives and false negatives, respectively.

A separate class-agnostic matching procedure was used for the semantic-head diagnostics of YOLO26-OBB-HMS. The confidence ordering and geometric IoU threshold were kept unchanged, but class labels were ignored during matching. This design preserves semantic errors for analysis when an object is localized correctly but assigned an incorrect category. Only geometrically matched detection–ground-truth pairs were included in the head-level confusion matrices.

Leaf-level predictions were taken directly from the native detector output, whereas intermediate-level and multi-class coarse-level predictions were obtained by applying argmax to the corresponding auxiliary head logits. Because all semantic heads within a given YOLO26-OBB-HMS model were evaluated using the same selected detections and identical geometric matches, parent-level performance was measured directly from the model’s intermediate and coarse predictions rather than inferred by mapping predicted leaf labels through the predefined taxonomy.

Table 5 shows that semantic accuracy generally increases as the prediction level moves from fine-grained leaf classes toward broader taxonomic categories, although the interpretation of the coarse level differs between the two datasets. On HRSC2016, YOLO26n-OBB-HMS achieves 87.84% leaf-level accuracy and 99.30% intermediate-level accuracy over 855 geometrically matched objects, while the corresponding s-scale model reaches 92.39% and 99.88% over 854 matches. The higher intermediate-level accuracies are consistent with the hierarchical organization of the retained taxonomy: several visually similar leaf categories share the same intermediate parent, so a prediction that is incorrect at the fine-grained level may still remain correct at the intermediate level. The s-scale model therefore provides a clear improvement over the n-scale variant at the leaf level (+4.55 pp), while both models approach saturation at the intermediate level.

The HRSC2016 coarse-level values require a different interpretation. For YOLO26-OBB-HMS, the coarse head is a single Ship-versus-background sigmoid output rather than a multi-class taxonomic classifier. Because the matched-object cohort contains only successfully localized ship instances, the reported values of 54.04% for the n-scale model and 68.85% for the s-scale model represent the proportion of matched ships whose auxiliary root score exceeds the fixed 0.50 threshold. They should therefore be interpreted as root-level acceptance rates conditional on localization, rather than as overall coarse-category classification accuracy or ship-versus-background detection performance. Importantly, this auxiliary coarse output does not gate, rerank, recalibrate, or otherwise alter the native leaf-level detection scores during inference.

On ShipRSImageNet, where the retained taxonomy contains two explicit coarse-level domains, the hierarchy-aware models exhibit consistently high accuracy across all semantic levels. YOLO26n-OBB-HMS achieves 90.75% leaf, 93.06% intermediate, and 99.91% coarse accuracy over 1167 matched objects, whereas YOLO26s-OBB-HMS reaches 93.78%, 95.29%, and 100.00%, respectively, over 1125 matches. The improvement from leaf to intermediate and coarse levels again reflects the progressively broader semantic grouping of the taxonomy, while the near-perfect coarse-level results indicate that errors among successfully localized objects occur predominantly within, rather than across, the broadest retained taxonomic groups.

HMS-Net provides an additional hierarchy-aware reference within the same matched-object analysis. On HRSC2016, its leaf-level accuracy of 88.54% lies between the n- and s-scale YOLO26-OBB-HMS variants, while its intermediate-level accuracy (99.30%) matches the n-scale hierarchy-aware model. On ShipRSImageNet, HMS-Net achieves 90.92% leaf, 94.93% intermediate, and 100.00% coarse accuracy over 1145 matched objects. These comparisons should nevertheless be interpreted descriptively because the number of geometrically matched objects is not identical across models and the metrics are conditioned on successful localization. Accordingly, Table 5 primarily characterizes the semantic behavior of each hierarchy-aware model on its localized-object cohort rather than providing an end-to-end detection ranking.

Table 6 provides a complementary view of the directly predicted hierarchical semantic heads by reporting support-weighted precision, recall, and F1 scores over the same geometrically matched object cohorts used in Table 5. In this single-label setting, support-weighted recall is numerically equivalent to classification accuracy, whereas the reported weighted F1 score is computed as the support-weighted average of the class-specific F1 scores and should not be interpreted as the harmonic mean of the reported weighted precision and recall.

On HRSC2016, YOLO26n-OBB-HMS achieves a leaf-level weighted precision, recall, and F1 score of 88.63%, 87.84%, and 87.90%, respectively, while the corresponding s-scale model reaches 92.37%, 92.39%, and 92.15%. The improvement across all three measures indicates more consistent fine-grained semantic classification for the s-scale model on the matched-object cohort. At the intermediate level, both models approach saturation: the n-scale model records 99.30% for all three metrics, whereas the s-scale variant reaches 99.88%. This pattern is consistent with the coarser semantic grouping of the retained taxonomy, where multiple fine-grained leaf classes share a common intermediate parent.

The HRSC2016 coarse-head metrics require a different interpretation. The reported precision is 100.00% for both model scales, while recall increases from 54.04% for YOLO26n-OBB-HMS to 68.85% for YOLO26s-OBB-HMS, yielding weighted F1 scores of 70.16% and 81.55%, respectively. However, because the matched-object cohort contains only positive ship instances, precision and background-rejection behavior are not informative for this auxiliary Ship-versus-background root output. The coarse-level recall therefore primarily reflects the proportion of successfully localized ships whose auxiliary root score exceeds the fixed decision threshold, rather than an end-to-end measure of coarse detection or background discrimination.

On ShipRSImageNet, where the retained taxonomy contains explicit multi-class intermediate and coarse levels, the hierarchy-aware models exhibit high and internally consistent precision, recall, and F1 scores across the hierarchy. YOLO26n-OBB-HMS achieves 90.99%, 90.75%, and 90.57% at the leaf level; 93.37%, 93.06%, and 93.03% at the intermediate level; and 99.91% for all three metrics at the coarse level. The corresponding s-scale model improves these values to 94.13%, 93.78%, and 93.74% at the leaf level; 95.45%, 95.29%, and 95.23% at the intermediate level; and 100.00% at the coarse level. The progressive increase from leaf to intermediate and coarse levels indicates that most remaining semantic errors among localized objects occur between closely related fine-grained categories rather than across the broadest taxonomic divisions.

Taken together, the results in Table 6 reinforce the matched-object findings of Table 5 while providing a more detailed view of class-imbalanced semantic performance. The close agreement among weighted precision, recall, and F1 at the leaf and intermediate levels suggests that the reported accuracies are not driven solely by a small subset of dominant classes. Nevertheless, because all metrics are computed only over geometrically matched detections, they should be interpreted as semantic performance conditional on successful localization rather than as substitutes for end-to-end detection AP.

Table 7 summarizes the localization coverage underlying the matched-object semantic analysis and therefore provides essential context for interpreting Table 5 and Table 6. On HRSC2016, YOLO26n-OBB-HMS matches 855 of the 963 retained ground-truth objects, corresponding to a coverage of 88.79%, while YOLO26s-OBB-HMS matches 854 objects and achieves a nearly identical coverage of 88.68%. The two models therefore expose their semantic heads to comparably sized localized-object cohorts on this dataset, with 108 and 109 missed ground-truth instances, respectively. The numbers of unmatched detections are likewise similar, at 134 for the n-scale model and 115 for the s-scale model.

On ShipRSImageNet, coverage is lower for both model scales. YOLO26n-OBB-HMS matches 1167 of the 1403 retained ground-truth objects, yielding 83.18% coverage, whereas YOLO26s-OBB-HMS matches 1125 objects, corresponding to 80.19% coverage. The n-scale model therefore provides a larger matched-object cohort despite the higher semantic accuracies observed for the s-scale model in Table 5 and Table 6. Specifically, the n-scale model leaves 236 ground-truth objects unmatched, compared with 278 for the s-scale model, while the numbers of unmatched detections are 469 and 266, respectively. These differences show that semantic accuracy and localization coverage capture distinct aspects of model behavior and should not be conflated.

Because the matching procedure is class-agnostic, a successfully matched detection indicates only that the object has been localized with sufficient geometric overlap; it does not imply that the predicted semantic label is correct. Conversely, ground-truth objects that are not geometrically matched are excluded from the semantic-head evaluation altogether. The head-level accuracies and weighted precision, recall, and F1 scores reported in Table 5 and Table 6 should therefore be interpreted strictly as semantic performance conditional on successful localization rather than as end-to-end detection performance.

Overall, Table 7 indicates that the HRSC2016 matched-object analysis is based on similarly covered cohorts for the n- and s-scale variants, whereas the ShipRSImageNet analysis involves a noticeable difference in localization coverage between model scales. This distinction is particularly important when comparing the higher semantic-head scores of YOLO26s-OBB-HMS with those of the n-scale model: the s-scale variant achieves stronger conditional semantic accuracy on a smaller set of successfully localized objects. Accordingly, the matched-object results should be read jointly with the coverage statistics rather than interpreted as unconditional model-level accuracy.

**Figure 3:**
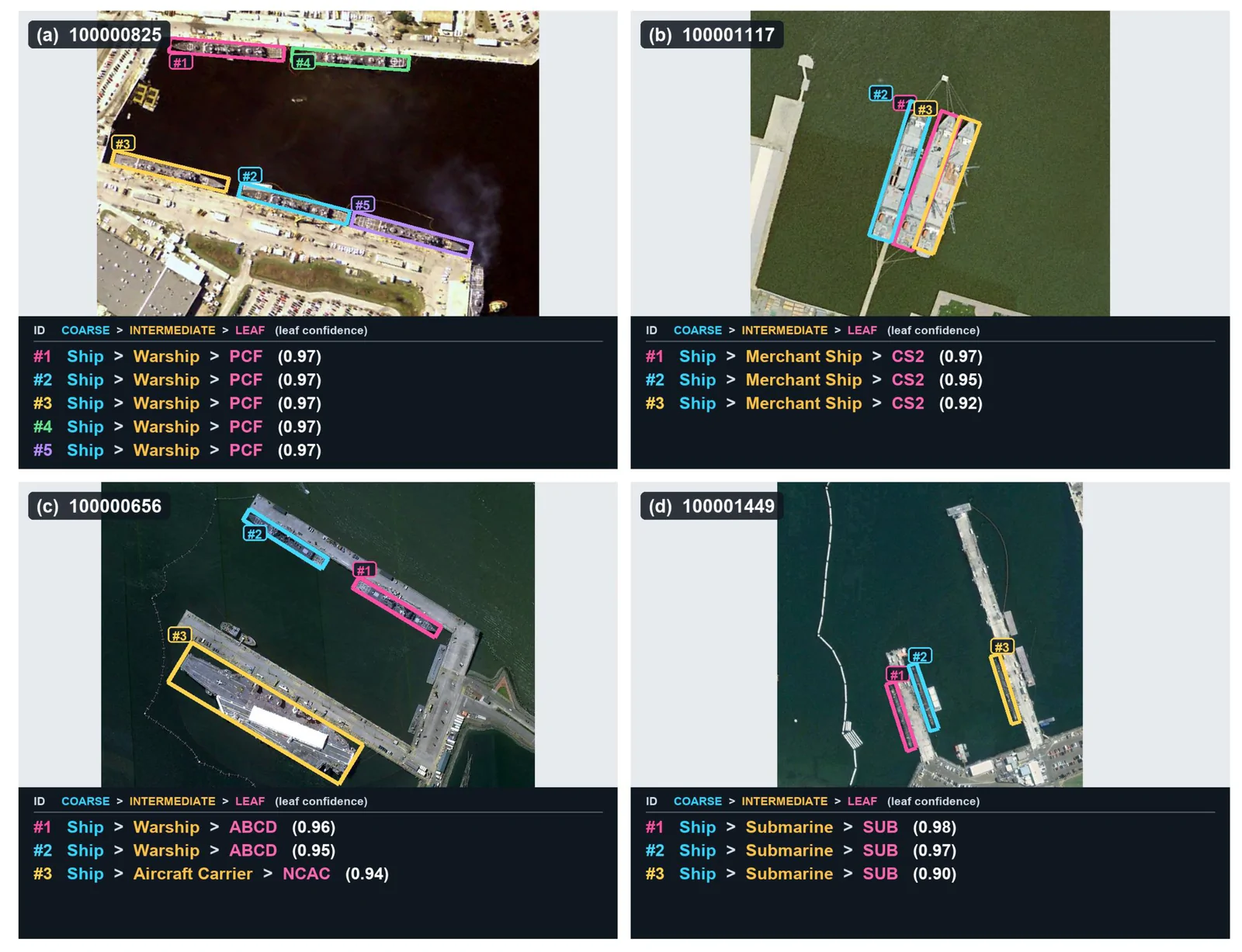
Qualitative results on HRSC2016. The four panels show numbered oriented detections together with their corresponding coarse-to-leaf hierarchical prediction paths.

Figure 3 links four representative HRSC2016 oriented detections to their numbered coarse-to-leaf prediction paths.

**Figure 4:**
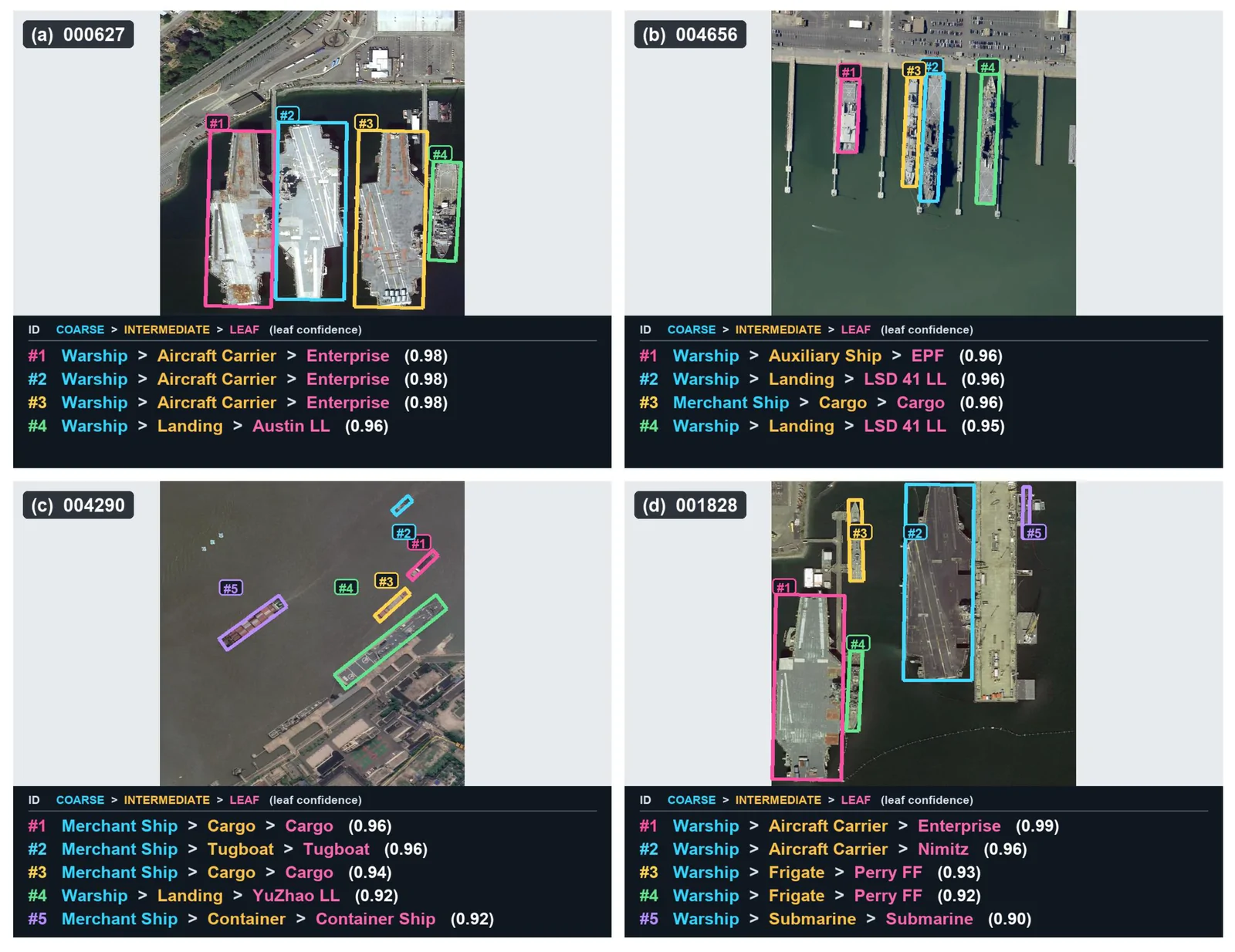
Qualitative results on ShipRSImageNet. The four panels show numbered oriented detections together with their corresponding coarse-to-leaf hierarchical prediction paths.

Figure 4 presents four ShipRSImageNet scenes with numbered detections and their corresponding hierarchical prediction paths.

### 4.7. Computational Efficiency and Runtime Performance

Computational efficiency was evaluated for YOLO26n-OBB, YOLO26s-OBB, and their corresponding HMS variants using the frozen checkpoints selected according to the predefined model-selection procedure. Runtime evaluation was performed on the HRSC2016 test partition.

All experiments were conducted on an NVIDIA GeForce RTX 4060 Ti GPU (NVIDIA Corporation, Santa Clara, CA, USA) with 16 GB of memory using PyTorch 2.13.0+cu130, CUDA 13.0, and Ultralytics (v8.4.12). Inference was executed with the native PyTorch backend in FP32 precision, using a batch size of 1, eight CPU threads, and zero data-loader workers. The nominal input size was set to 640 pixels; however, aspect-ratio-preserving rectangular validation preprocessing was employed rather than resizing every image to a fixed 640 × 640 tensor. The detection confidence threshold was set to 0.001, with a maximum of 300 detections per image. Test-time augmentation and model compilation were disabled, whereas standard backend fusion and the built-in warm-up procedure were applied before timing.

Each frozen checkpoint was evaluated over three complete inference passes. Table 8 reports the mean forward latency and corresponding sample standard deviation across these runs. CUDA synchronization was performed at the timing boundaries to ensure reliable GPU measurements. The reported forward latency includes framework dispatch, model forward execution, native decoding, and candidate selection, while excluding image preprocessing, data loading, metric accumulation, visualization, and validator-level post-processing. Accordingly, the reported latency should be interpreted as forward-pass latency rather than end-to-end inference latency.

Forward FPS was calculated as 1000 divided by the unrounded mean forward latency in milliseconds and therefore represents reciprocal forward-pass throughput rather than complete end-to-end system throughput. Within Table 8, ΔmAP50 denotes the difference between each hierarchy-aware model and its scale-matched native baseline under the same runtime evaluation protocol. Parameter counts correspond to the fused retained model graph and include parameters associated with retained auxiliary components, even when these components do not directly contribute to final leaf-level detection ranking. The reported mAP50 values were obtained from the frozen checkpoints using the native OBB evaluator on the HRSC2016 test partition under the same runtime evaluation protocol and should therefore be interpreted specifically within this evaluation setting.

At the n scale, YOLO26n-OBB-HMS increases mAP50 from 73.11% to 78.84%, corresponding to an improvement of 5.73 percentage points. This accuracy gain is accompanied by an increase in mean forward latency from 19.24 to 32.87 ms, representing a 70.8% increase, while reciprocal forward throughput decreases from 52.0 to 30.4 FPS. At the s scale, YOLO26s-OBB-HMS increases mAP50 from 81.95% to 82.86%, corresponding to a gain of 0.91 percentage points. Mean forward latency simultaneously increases from 19.45 to 32.98 ms (+69.6%), with forward throughput decreasing from 51.4 to 30.3 FPS. These results indicate that the HMS formulation improves detection accuracy at both model scales, although the magnitude of the accuracy gain differs substantially between the n- and s-scale variants and is accompanied by increased computational cost.

The measured overhead corresponds to the complete, unpruned HMS inference graph. In the retained architecture, the Axis-wise Context Block (ACB) remains active before pyramid fusion, while the one-to-one detection branch retains six Feature Guidance (FG) transformations distributed across its detection scales, together with the parent-level prediction components and semantic embeddings. By contrast, Poincaré-distance computations are used exclusively for training-time supervision and do not participate in leaf-level detection ranking during inference.

The runtime measurements reported in Table 8 characterize the complete models rather than individual architectural components. Neither the six FG modules nor the auxiliary prediction components were independently timed. Although the corresponding ablation experiments provide matched accuracy comparisons, they do not provide module-specific runtime measurements. Consequently, the observed increase in forward latency cannot be quantitatively attributed to ACB, FG, or the auxiliary hierarchical components individually. Any such decomposition would require dedicated component-level profiling or matched runtime ablations.

Computational complexity was additionally quantified using PyTorch FlopCounterMode after backend fusion and with the models operating in evaluation mode. FLOPs were measured using an FP32 input tensor of size 1 × 3 × 640 × 640, with each multiply–accumulate operation counted as two floating-point operations. The reported estimates include convolutional operations, linear projections, matrix multiplications, and attention products. Operator tracing and analytical verification were additionally employed to ensure that the computations associated with all six FG modules were included.

The reported FLOP estimates intentionally exclude element-wise operations, activation functions, normalization, softmax, pooling, interpolation, decoding arithmetic, memory movement, and indexing operations. Moreover, GFLOPs were calculated using a fixed 640 × 640 input tensor, whereas empirical latency was measured using aspect-ratio-preserving rectangular validation inputs. The reported GFLOP values and measured latency therefore characterize different computational conditions, and their ratio should not be interpreted as achieved hardware throughput.

YOLO26n-OBB-HMS requires 10.78 GFLOPs and 4.776 M parameters, compared with 5.58 GFLOPs and 2.451 M parameters for the native YOLO26n-OBB model. This corresponds to increases of approximately 93.2% in arithmetic complexity and 94.9% in parameter count. Similarly, YOLO26s-OBB-HMS requires 41.70 GFLOPs and 18.954 M parameters, compared with 21.79 GFLOPs and 9.759 M parameters for YOLO26s-OBB, corresponding to increases of approximately 91.4% and 94.2%, respectively. Thus, the retained HMS architecture approximately doubles both arithmetic complexity and parameter count at both model scales, while the measured forward latency increases by approximately 70%. This difference further demonstrates that theoretical operation counts should not be interpreted as a direct predictor of empirical GPU latency.

## 5. Conclusions

This study introduced YOLO26-OBB-HMS, a hierarchy-aware extension of YOLO26-OBB that integrates axis-wise contextual modeling, local cross-level Feature Guidance, assignment-aligned parent supervision, and training-time Poincaré path supervision while preserving the detector’s native oriented-box formulation and leaf-score inference pathway. HRSC2016 was evaluated on the filtered official test partition after internal model selection, whereas ShipRSImageNet was evaluated on the prepared final-evaluation partition, which was kept separate from the prepared validation partition used for model selection.

Across five paired HRSC2016 ablation runs, the selected H7 configuration achieved 77.42 ± 0.18% mAP50 and 67.46 ± 0.33% mAP50:95, compared with 73.07 ± 0.07% and 66.14 ± 0.22% for the native Y0 baseline. H7 also exceeded the matched Euclidean control H13 by 1.73 and 1.63 percentage points, respectively. Holm-adjusted paired analyses within the defined five-run, seed-matched protocol supported the H7/Y0 and H7/H13 differences for both metrics. In contrast, the H7/H10 FG comparison did not cross the conventional 0.05 significance threshold for either metric, and the direction of the FG effect reversed when parent supervision was disabled. These findings indicate that the benefit of FG depends on the broader hierarchical-supervision configuration rather than representing a configuration-independent effect. Accordingly, the statistical findings are interpreted within the evaluated five-run protocol and are not generalized beyond datasets, model scales, or experimental conditions for which equivalent repeated-run evidence is unavailable.

Under the common class-wise AP50 evaluation protocol, the n- and s-scale hierarchy-aware models achieved 77.5%/82.7% on HRSC2016 and 75.4%/80.6% on ShipRSImageNet, compared with 73.1%/81.7% and 74.1%/80.2% for their corresponding native YOLO26-OBB models. The s-scale hierarchy-aware model also exceeded the HMS-Net reference under the common prepared evaluation protocol, although these cross-framework differences remain descriptive because the compared detector families retain distinct training and implementation pipelines. Class-specific gains and regressions further indicate that the aggregate improvements are not uniformly distributed across the retained taxonomy.

These accuracy gains are accompanied by substantial computational overhead. At the n scale, the hierarchy-aware model nearly doubles both parameter count and GFLOPs, increases forward latency by 70.8%, and reduces reciprocal forward throughput from 52.0 to 30.4 FPS. The present formulation should therefore be regarded as accuracy-oriented rather than deployment-optimized. Future work should investigate capacity-matched attention controls; sensitivity to ACB kernel size, FG window size, and input resolution; broader multi-parameter and cross-dataset robustness of the selected Poincaré operating point; multi-seed replication across datasets and model scales; verified native head-level baselines and hyperbolic-geometry diagnostics; and deployment-pruned inference graphs.

Finally, because the ShipRSImageNet experiments use a prepared repartition rather than the original official split and earlier exploratory work had access to imagery from the original validation material, the reported ShipRSImageNet results should not be interpreted as independent official-test benchmark performance.

## Figures and Tables

**Table 1:** HRSC2016 ablation configuration matrix.

ID	ACB	MRFR	FG	Parent	Proxy	Path	Variant
Y0	—	—	—	—	—	—	Native YOLO
H0	—	✓	✓	✓	✓	—	HMS reference
H1	✓	✓	✓	✓	✓	—	H0 + ACB
H2	✓	—	✓	✓	✓	—	H1 − MRFR
H3	✓	✓	✓	✓	—	—	H1 − Proxy
H4	✓	✓	✓	—	✓	—	H1 − Parent
H5	—	—	✓	✓	✓	—	H0 − MRFR
H6	—	—	✓	✓	—	Poincaré	Proxy → path CE + Tax
H7	✓	—	✓	✓	—	Poincaré	H6 + ACB
H8	—	✓	✓	✓	—	Poincaré	H6 + MRFR
H9	✓	—	✓	✓	—	—	H7 − CE and Tax
H10	✓	—	—	✓	—	Poincaré	H7 − FG
H11	✓	—	✓	✓	—	Poincaré	Path CE only
H12	✓	—	✓	✓	—	Poincaré	Taxonomy only
H13	✓	—	✓	✓	—	Euclidean	Metric swap; CE + Tax kept
H14	✓	—	✓	—	—	Poincaré	Parent gain = 0
H15	✓	—	—	—	—	Poincaré	H14 − FG

Note: ✓ indicates that the corresponding component is ena-bled, whereas — indicates that it is disabled.

**Table 2:** HRSC2016 n-scale ablation on the filtered official test set. Values are percentages.

ID	mAP50 Mean ± SD (%)	mAP50 [Min–Max] (%)	mAP50:95 Mean ± SD (%)	mAP50:95 [Min–Max] (%)
Y0	73.07 ± 0.07	[72.95–73.14]	66.14 ± 0.22	[65.85–66.38]
H0	68.00 ± 0.21	[67.76–68.25]	55.74 ± 0.19	[55.56–56.04]
H1	60.85 ± 0.36	[60.52–61.42]	50.22 ± 0.07	[50.15–50.32]
H2	64.82 ± 0.26	[64.40–65.05]	50.70 ± 0.21	[50.48–50.98]
H3	74.86 ± 0.33	[74.40–75.17]	64.79 ± 0.24	[64.44–64.98]
H4	65.72 ± 0.08	[65.61–65.80]	53.04 ± 0.09	[52.91–53.15]
H5	68.20 ± 0.23	[67.82–68.41]	56.33 ± 0.27	[56.04–56.66]
H6	76.02 ± 0.24	[75.77–76.42]	66.08 ± 0.10	[65.95–66.23]
H7	77.42 ± 0.18	[77.17–77.64]	67.46 ± 0.33	[67.11–67.96]
H8	76.61 ± 0.14	[76.48–76.80]	66.09 ± 0.09	[65.95–66.17]
H9	77.28 ± 0.15	[77.02–77.38]	66.63 ± 0.03	[66.61–66.68]
H10	76.63 ± 0.27	[76.22–76.90]	66.93 ± 0.17	[66.79–67.16]
H11	77.36 ± 0.09	[77.25–77.49]	66.93 ± 0.07	[66.85–67.02]
H12	74.17 ± 0.24	[73.92–74.55]	65.67 ± 0.15	[65.46–65.80]
H13	75.69 ± 0.12	[75.51–75.80]	65.83 ± 0.28	[65.50–66.16]
H14	73.33 ± 0.22	[73.11–73.58]	64.65 ± 0.34	[64.16–65.00]
H15	76.21 ± 0.22	[76.03–76.45]	67.45 ± 0.12	[67.32–67.58]

**Table 3:** Class-wise AP50 comparison on the filtered HRSC2016 test set using a common COCO-style evaluation. Values are percentages.

Class	Oriented R-CNN	RTMDet-R-s	HMS-Net	YOLO26n-OBB	YOLO26n-OBB-HMS	YOLO26s-OBB	YOLO26s-OBB-HMS
AKAC	100.0	50.0	99.5	99.5	99.5	99.5	99.5
ECAC	50.4	59.1	69.3	48.9	59.3	51.0	51.2
MCAC	99.2	90.2	99.5	95.4	99.5	99.5	99.5
NCAC	78.1	80.4	89.2	81.2	89.9	84.4	86.5
ABCD	86.9	91.3	91.0	84.3	89.6	89.4	91.7
AATD	82.2	76.2	70.5	70.9	62.7	79.2	73.1
LUT	74.8	51.4	68.2	74.5	68.2	74.5	68.2
MS	95.0	90.9	93.2	84.1	97.2	92.2	96.8
OXO	75.3	61.9	77.9	55.5	72.7	76.8	82.7
PCF	84.5	89.2	82.6	72.9	84.2	82.8	86.4
SATD	89.2	85.8	46.2	53.6	41.3	81.9	89.8
TAS	89.7	93.4	93.0	90.7	91.0	93.1	96.6
TCC	87.9	91.7	80.5	77.9	86.2	87.8	89.8
WILD	76.9	76.6	66.6	49.0	46.4	76.0	71.5
CC1	86.7	83.4	79.8	71.4	71.1	87.5	84.8
CC2	85.0	81.4	86.4	88.9	96.1	88.4	98.1
CS	65.0	54.9	64.8	53.6	66.5	54.9	52.9
CS2	76.6	79.1	84.8	84.5	86.5	85.2	76.7
CRU	100.0	70.6	42.2	61.5	89.5	99.5	99.5
HVR	67.7	56.8	51.5	56.5	47.8	46.3	48.9
SUB	55.9	77.6	81.3	79.8	81.4	86.0	91.9
mAP50 (%)	81.3	75.8	77.0	73.1	77.5	81.7	82.7

**Table 4:** Class-wise AP50 comparison on the prepared ShipRSImageNet final-evaluation partition using a common COCO-style evaluation. Values are percentages.

Class	Oriented R-CNN	RTMDet-R-s	HMS-Net	YOLO26n-OBB	YOLO26n-OBB-HMS	YOLO26s-OBB	YOLO26s-OBB-HMS
Submarine	65.7	68.3	88.0	89.2	89.9	93.3	94.4
Enterprise	83.2	84.4	86.9	84.1	79.9	88.5	72.1
Nimitz	87.2	76.9	95.0	92.4	92.5	96.8	91.0
Midway	83.3	93.3	92.8	81.8	92.8	92.8	99.5
Ticonderoga	84.3	83.1	78.0	84.7	79.7	82.1	81.8
Atago DD	66.9	65.8	79.6	62.2	64.5	72.5	66.4
Arleigh Burke DD	90.1	91.3	91.2	94.6	91.4	88.4	95.3
Hatsuyuki DD	61.8	62.3	53.6	58.6	67.4	58.4	60.4
Hyuga DD	96.7	99.6	96.1	98.8	95.1	90.2	95.1
Asagiri DD	47.1	64.2	42.8	22.5	43.9	71.8	74.4
Perry FF	80.3	84.7	89.1	83.3	83.3	92.5	90.6
YuTing LL	73.9	84.3	88.0	80.1	67.7	86.5	89.5
YuDeng LL	45.5	46.0	88.9	83.6	83.6	88.0	79.3
YuDao LL	100.0	83.6	84.5	73.2	99.5	89.5	96.2
YuZhao LL	100.0	95.2	99.5	99.5	89.8	94.8	99.5
Austin LL	65.7	64.9	84.0	83.5	81.6	82.7	62.1
Osumi LL	100.0	100.0	99.5	99.5	93.1	97.2	99.5
Wasp LL	87.3	91.0	97.2	92.4	99.5	99.5	99.5
LSD 41 LL	71.7	67.5	75.8	68.4	63.0	86.9	84.8
LHA LL	81.3	89.5	98.5	93.2	96.1	97.3	91.5
Commander	91.9	86.0	82.6	74.0	82.3	88.2	90.9
Medical Ship	84.4	82.2	79.5	86.6	53.8	71.5	79.5
Test Ship	52.2	55.6	58.1	63.8	61.7	66.7	81.6
Training Ship	92.3	91.7	98.1	87.3	87.2	94.8	99.5
AOE	81.5	81.5	77.9	67.9	76.1	87.3	99.5
Masyuu AS	95.8	87.3	87.7	78.6	89.2	89.7	94.5
Sanantonio AS	79.6	81.7	56.7	62.3	79.2	81.4	83.3
EPF	89.0	90.0	89.5	89.5	89.5	89.5	81.5
Container Ship	55.9	56.8	70.5	67.7	58.9	63.4	62.5
RoRo	85.6	87.8	88.5	83.4	83.4	84.5	85.9
Cargo	54.0	53.3	70.5	69.2	72.9	68.3	67.1
Barge	19.6	21.5	31.3	26.7	25.0	26.2	29.9
Tugboat	45.7	45.6	54.5	47.0	47.7	54.0	43.6
Ferry	39.6	27.5	39.4	34.5	27.6	46.3	40.0
Yacht	64.2	64.8	79.7	72.9	77.8	81.1	83.4
Oil Tanker	24.2	48.9	64.3	56.1	64.8	64.8	67.1
Hovercraft	63.1	75.0	59.6	47.1	59.8	60.6	68.3
mAP50 (%)	72.7	73.9	78.3	74.1	75.4	80.2	80.6

**Table 5:** Hierarchical classification accuracy on matched objects. Values are percentages; (M) denotes matched objects.

Dataset	Model	M	Leaf Accuracy	Middle Accuracy	Coarse Accuracy
HRSC2016	YOLO26n-OBB-HMS	855	87.84	99.30	54.04
HRSC2016	YOLO26s-OBB-HMS	854	92.39	99.88	68.85
HRSC2016	HMS-Net	855	88.54	99.30	63.39
ShipRSImageNet	YOLO26n-OBB-HMS	1167	90.75	93.06	99.91
ShipRSImageNet	YOLO26s-OBB-HMS	1125	93.78	95.29	100.00
ShipRSImageNet	HMS-Net	1145	90.92	94.93	100.00

**Table 6:** Support-weighted precision, recall, and F1 scores for the directly predicted hierarchical semantic heads. Values are percentages.

Dataset	Model	Head	Precision	Recall	F1-Score
HRSC2016	YOLO26n-OBB-HMS	Leaf	88.63	87.84	87.90
HRSC2016	YOLO26n-OBB-HMS	Middle	99.30	99.30	99.30
HRSC2016	YOLO26n-OBB-HMS	Coarse	100.00	54.04	70.16
HRSC2016	YOLO26s-OBB-HMS	Leaf	92.37	92.39	92.15
HRSC2016	YOLO26s-OBB-HMS	Middle	99.88	99.88	99.88
HRSC2016	YOLO26s-OBB-HMS	Coarse	100.00	68.85	81.55
ShipRSImageNet	YOLO26n-OBB-HMS	Leaf	90.99	90.75	90.57
ShipRSImageNet	YOLO26n-OBB-HMS	Middle	93.37	93.06	93.03
ShipRSImageNet	YOLO26n-OBB-HMS	Coarse	99.91	99.91	99.91
ShipRSImageNet	YOLO26s-OBB-HMS	Leaf	94.13	93.78	93.74
ShipRSImageNet	YOLO26s-OBB-HMS	Middle	95.45	95.29	95.23
ShipRSImageNet	YOLO26s-OBB-HMS	Coarse	100.00	100.00	100.00

**Table 7:** Localization coverage for the matched-object classification cohort.

Dataset	Model	Retained Ground Truth	Matched	Missed Ground Truth	Unmatched Detections	Coverage (%)
HRSC2016	YOLO26n-OBB-HMS	963	855	108	134	88.79
HRSC2016	YOLO26s-OBB-HMS	963	854	109	115	88.68
ShipRSImageNet	YOLO26n-OBB-HMS	1403	1167	236	469	83.18
ShipRSImageNet	YOLO26s-OBB-HMS	1403	1125	278	266	80.19

**Table 8:** Computational complexity and runtime performance on HRSC2016. Parameters are reported after backend fusion; GFLOPs use a 640 × 640 input, and latency is mean ± SD over three complete inference passes.

Model	Parameters (M)	GFLOPs	mAP50 (%)	∆mAP50 (pp)	Latency (ms)	Forward FPS
YOLO26n-OBB	2.451	5.58	73.11	-	19.24 ± 0.30	52.0
YOLO26n-OBB-HMS	4.776	10.78	78.84	+5.73	32.87 ± 0.23	30.4
YOLO26s-OBB	9.759	21.79	81.95	-	19.45 ± 0.18	51.4
YOLO26s-OBB-HMS	18.954	41.70	82.86	+0.91	32.98 ± 0.53	30.3

## Data Availability

The HRSC2016 [25] and ShipRSImageNet [26] datasets are available from their respective cited sources. Supplementary Tables S1 and S2 provide the prepared taxonomy mappings, retained-class annotation counts, and dataset-preparation information. Supplementary Table S3 reports the individual results of the five HRSC2016 ablation runs and their corresponding random seeds (0, 42, 100, 120, and 150), while Supplementary Table S4 presents the paired statistical comparisons and Holm-adjusted *p*-values. Table S2 reports both Test GT (official-test support) and All prepared GT (train + validation + test). The exact image-ID manifest of the internal selection subset and the selected checkpoint epochs are not included in the accompanying material.

## Supplementary Materials

The following supporting information can be downloaded at https://www.mdpi.com/article/10.3390/s26206584/s1, Table S1. ShipRSImageNet taxonomy and annotation audit. Table S2. HRSC2016 taxonomy and retained-class counts. Table S3. HRSC2016 ablation runs. Table S4. HRSC2016 paired ablation contrasts.

## Abbreviations

The following class codes are used for the retained HRSC2016 leaves in Table 3. The code-to-class correspondence follows the typed-ship protocol in [9].

Code	Full class name
AKAC	Admiral Kuznetsov aircraft carrier
ECAC	Enterprise-class aircraft carrier
MCAC	Midway-class aircraft carrier
NCAC	Nimitz-class aircraft carrier
ABCD	Arleigh Burke-class destroyer
AATD	Austin-class amphibious transport dock
LUT	Lute
MS	Medical ship
OXO	OXO
PCF	Perry-class frigate
SATD	San Antonio-class amphibious transport dock
TAS	Tarawa-class amphibious assault ship
TCC	Ticonderoga-class cruiser
WILD	Whidbey Island-class dock landing ship
CC1	Car carrier 1
CC2	Car carrier 2
CS	Container ship
CS2	Container ship 2
CRU	Cruise ship
HVR	Hovercraft
SUB	Submarine
