# Supplementary material for: Hierarchical YOLO26 with Axis-Wise Context Block and Poincaré Supervision for Fine-Grained Ship Detection in Remote Sensing
Source: Sensors (Basel). 2026 Oct 16;26(20):6584. doi: 10.3390/s26206584 (PMC13709215; doi:10.3390/s26206584)
Supplement: Supplementary file 1 [file sensors-26-06584-s001.zip › sensors-4588793-supplementary.pdf]

Supplementary Materials

Tables S1 and S2 document the prepared ShipRSImageNet and HRSC2016 taxonomies. Table S3 reports the five measured HRSC2016 ablation runs, while Table S4 presents the ten targeted, seed-matched contrasts together with the corresponding paired statistical tests and Holm-adjusted p-values. All AP values are percentages.

Table S1. ShipRSImageNet taxonomy and annotation audit.

S1a. The 37 retained leaves, 0-based code IDs, 1-based raw IDs, coarse and intermediate parents, unique GT support on the 550-image final evaluation partition, and aggregate counts across the prepared labeled partitions. The export total includes the duplicate record later removed by the validation loader.

Code	Raw	Leaf	Coarse	Intermediate	Final eval GT	Labeled export
0	1	Submarine	Warship	Submarine	171	837
1	2	Enterprise	Warship	Aircraft Carrier	18	87
2	3	Nimitz	Warship	Aircraft Carrier	19	103
3	4	Midway	Warship	Aircraft Carrier	5	18
4	5	Ticonderoga	Warship	Cruiser	77	335
5	6	Atago DD	Warship	Destroyer	41	208
6	7	Arleigh Burke DD	Warship	Destroyer	130	526
7	8	Hatsuyuki DD	Warship	Destroyer	22	110
8	9	Hyuga DD	Warship	Destroyer	16	86
9	10	Asagiri DD	Warship	Destroyer	14	62
10	11	Perry FF	Warship	Frigate	77	500
11	12	YuTing LL	Warship	Landing	19	80
12	13	YuDeng LL	Warship	Landing	11	64
13	14	YuDao LL	Warship	Landing	5	45
14	15	YuZhao LL	Warship	Landing	6	37
15	16	Austin LL	Warship	Landing	27	103
16	17	Osumi LL	Warship	Landing	6	34
17	18	Wasp LL	Warship	Landing	6	20
18	19	LSD 41 LL	Warship	Landing	21	116
19	20	LHA LL	Warship	Landing	31	157
20	21	Commander	Warship	Commander	32	120
21	22	Medical Ship	Warship	Auxiliary Ship	5	27
22	23	Test Ship	Warship	Auxiliary Ship	7	50
23	24	Training Ship	Warship	Auxiliary Ship	11	42

S1a continued. Retained ShipRSImageNet leaf categories.

Code	Raw	Leaf	Coarse	Intermediate	Final eval GT	Labeled export
24	25	AOE	Warship	Auxiliary Ship	11	48
25	26	Masyuu AS	Warship	Auxiliary Ship	8	36
26	27	Sanantonio AS	Warship	Auxiliary Ship	13	61
27	28	EPF	Warship	Auxiliary Ship	10	52
28	29	Container Ship	Merchant Ship	Container	71	304
29	30	RoRo	Merchant Ship	RoRo	20	127
30	31	Cargo	Merchant Ship	Cargo	169	826
31	32	Barge	Merchant Ship	Barge	22	183
32	33	Tugboat	Merchant Ship	Tugboat	46	243
33	34	Ferry	Merchant Ship	Ferry	53	244
34	35	Yacht	Merchant Ship	Yacht	140	641
35	36	Oil Tanker	Merchant Ship	Oil Tanker	32	161
36	37	Hovercraft	Merchant Ship	Hovercraft	31	260
		Total			1403	6953

Following the dataset preparation and repartitioning described in Section 4.1, the prepared ShipRSImageNet training partition contains 4,458 retained annotation entries in 2,198 images. The prepared 550-image final-evaluation partition contains 1,404 retained XML annotation entries. The Ultralytics loader removes one duplicate Container Ship entry in image 1907__2012_1840, leaving 1,403 unique ground-truth objects for evaluation. The “Labeled export” column aggregates the 4,458 training entries, 1091 validation entries and 1,404 final-evaluation entries, totaling 6,953 entries before duplicate removal.

S1b. Excluded categories in the prepared task. These annotations are removed rather than treated as ignore regions.

Original category	Final eval removed	Labeled export removed
Other Ship	297	1347
Other Warship	209	1171
Dock	154	898
Other Landing	18	87
Other Auxiliary Ship	18	78
Other Destroyer	46	217
Other Frigate	28	168
Other Aircraft Carrier	4	34
Motorboat	398	1588
Other Merchant	50	200
Fishing Vessel	99	417
Sailboat	341	666
Patrol	37	139
Total	1699	7010

Table S2. HRSC2016 taxonomy and retained-class counts.

The original HMS-Net study combines official training (436 images) and validation (181 images) for development and evaluates on official test (444 images). Our filtered preparation contains 395/175/404 images and 882/426/963 objects in those partitions. Ablations use the 570-image train+validation pool (1,308 objects) for training and the 404-image filtered official test (963 objects) for evaluation. Table S2 now reports both Test GT (official test support) and All prepared GT (train+val+test). Exact image-ID manifests and per-split class supports are released under docs/reproducibility/.

Code	Raw	Original HRSC ID	Leaf	Intermediate	Test GT	All prepared GT
0	1	100000013	AKAC	Aircraft Carrier	2	10
1	2	100000006	ECAC	Aircraft Carrier	13	33
2	3	100000032	MCAC	Aircraft Carrier	10	23
3	4	100000005	NCAC	Aircraft Carrier	38	88
4	5	100000007	ABCD	Warship	164	430
5	6	100000015	AATD	Warship	37	88
6	7	100000028	LUT	Warship	4	9
7	8	100000029	MS	Warship	10	25
8	9	100000019	OXO	Warship	42	89
9	10	100000009	PCF	Warship	133	265
10	11	100000010	SATD	Warship	22	52
11	12	100000016	TAS	Aircraft Carrier	50	149
12	13	100000011	TCC	Warship	110	270
13	14	100000008	WILD	Warship	42	108
14	15	100000020	CC1	Merchant Ship	16	31
15	16	100000030	CC2	Merchant Ship	16	41
16	17	100000018	CS	Merchant Ship	18	52
17	18	100000025	CS2	Merchant Ship	122	289
18	19	100000026	CRU	Merchant Ship	4	10
19	20	100000022	HVR	Merchant Ship	50	105
20	21	100000027	SUB	Submarine	60	104
			Total		963	2271

All HRSC2016 leaves have the Ship coarse parent. The filtered development pool contains 1,308 retained objects and the filtered official test contains 963, for 2,271 total. Low-support test classes (e.g., AKAC, LUT, CRU) should be interpreted using the Test GT column rather than aggregate prepared counts alone.

Table S3. HRSC2016 ablation runs.

Each cell reports mAP50 / mAP50:95 (%) for one complete run. Runs 1–5 correspond to random seeds 0, 42, 100, 120, and 150, respectively. All rows use the fixed data split and checkpoint-selection rule described in Section 4.3.

ID	Run 1 (seed 0)	Run 2 (seed 42)	Run 3 (seed 100)	Run 4 (seed 120)	Run 5 (seed 150)
Y0	73.14 / 66.38	73.08 / 66.02	73.08 / 65.85	72.95 / 66.33	73.10 / 66.13
H0	68.25 / 55.56	67.76 / 56.04	68.17 / 55.59	67.87 / 55.72	67.95 / 55.80
H1	60.52 / 50.25	60.82 / 50.23	61.42 / 50.32	60.92 / 50.15	60.57 / 50.15
H2	65.05 / 50.52	65.00 / 50.84	64.76 / 50.98	64.89 / 50.48	64.40 / 50.69
H3	74.62 / 64.44	75.17 / 64.98	75.10 / 64.65	74.40 / 64.93	75.01 / 64.95
H4	65.77 / 52.91	65.80 / 53.15	65.65 / 53.07	65.77 / 53.00	65.61 / 53.07
H5	68.41 / 56.05	68.28 / 56.47	68.31 / 56.43	68.18 / 56.04	67.82 / 56.66
H6	75.77 / 65.95	76.42 / 66.04	76.01 / 66.07	75.98 / 66.11	75.92 / 66.23
H7	77.38 / 67.35	77.52 / 67.30	77.38 / 67.96	77.17 / 67.11	77.64 / 67.59
H8	76.80 / 65.95	76.48 / 66.14	76.51 / 66.04	76.55 / 66.16	76.71 / 66.17
H9	77.26 / 66.63	77.02 / 66.61	77.37 / 66.61	77.38 / 66.68	77.36 / 66.62
H10	76.90 / 66.79	76.60 / 67.07	76.22 / 67.16	76.84 / 66.85	76.59 / 66.79
H11	77.49 / 66.92	77.41 / 67.02	77.32 / 66.85	77.33 / 66.87	77.25 / 66.98
H12	74.02 / 65.80	74.20 / 65.56	74.14 / 65.46	74.55 / 65.78	73.92 / 65.75
H13	75.74 / 65.50	75.77 / 66.08	75.51 / 66.16	75.80 / 65.66	75.62 / 65.75
H14	73.11 / 64.16	73.56 / 64.45	73.18 / 64.84	73.23 / 64.80	73.58 / 65.00
H15	76.03 / 67.45	76.04 / 67.58	76.44 / 67.34	76.09 / 67.56	76.45 / 67.32

Table S4. HRSC2016 paired ablation contrasts.

Contrast	Metric	Mean difference (pp)	t(4)	Raw p	Holm-adjusted p
H7 − Y0	mAP50	+4.35	+70.6828	2.40058e-07	4.80116e-06
H7 − Y0	mAP50:95	+1.32	+5.7353	0.00457774	0.0411997
H7 − H6	mAP50	+1.40	+11.7842	0.00029675	0.003561
H7 − H6	mAP50:95	+1.38	+9.5403	0.000674119	0.00741531
H7 − H9	mAP50	+0.14	+1.1646	0.308886	0.617771
H7 − H9	mAP50:95	+0.83	+5.3612	0.00584177	0.0411997
H7 − H11	mAP50	+0.06	+0.5971	0.582569	0.617771
H7 − H11	mAP50:95	+0.53	+3.3796	0.0277954	0.111182
H7 − H12	mAP50	+3.25	+18.2524	5.29946e-05	0.000900908
H7 − H12	mAP50:95	+1.79	+9.0842	0.000814171	0.00814171
H7 − H10	mAP50	+0.79	+4.8449	0.00837058	0.0502235
H7 − H10	mAP50:95	+0.53	+4.2598	0.0130556	0.0652778
H14 − H15	mAP50	-2.88	-23.2490	2.02859e-05	0.000365147
H14 − H15	mAP50:95	-2.80	-15.2866	0.000106811	0.00149535
H7 − H13	mAP50	+1.73	+15.7338	9.53264e-05	0.0014299
H7 − H13	mAP50:95	+1.63	+12.8644	0.000210525	0.00273682
H7 − H14	mAP50	+4.09	+62.7799	3.85599e-07	7.32637e-06
H7 − H14	mAP50:95	+2.81	+17.1057	6.85107e-05	0.00109617
H10 − H15	mAP50	+0.42	+2.0755	0.106565	0.319694
H10 − H15	mAP50:95	-0.52	-5.5942	0.00501091	0.0411997

The signed mean difference is the left-hand configuration minus the right-hand configuration. The five aligned seed results were compared with two-sided paired t-tests (four degrees of freedom). Holm adjustment was applied to the full family of 20 raw p values from the ten displayed contrasts and two metrics. Calculations use the scores recorded to two decimal places in Table S3. These analyses are interpreted within the defined five-run, seed-matched protocol and do not test the FG-by-parent interaction..
